# Phylogeny‐guided characterization of glycosyltransferases for epothilone glycosylation

**DOI:** 10.1111/1751-7915.13421

**Published:** 2019-05-08

**Authors:** Peng Zhang, Zheng Zhang, Zhi‐feng Li, Qi Chen, Yao‐yao Li, Ya Gong, Xin‐jing Yue, Duo‐hong Sheng, You‐ming Zhang, Changsheng Wu, Yue‐zhong Li

**Affiliations:** ^1^ State Key Laboratory of Microbial Technology Institute of Microbial Technology Shandong University Qingdao 266237 China

## Abstract

Glycosylation of natural products can influence their pharmacological properties, and efficient glycosyltransferases (GTs) are critical for this purpose. The polyketide epothilones are potent anti‐tumour compounds, and YjiC is the only reported GT for the glycosylation of epothilone. In this study, we phylogenetically analysed 8261 GTs deposited in CAZy database and revealed that YjiC locates in a subbranch of the Macrolide I group, forming the YjiC‐subbranch with 160 GT sequences. We demonstrated that the YjiC‐subbranch GTs are normally efficient in epothilone glycosylation, but some showed low glycosylation activities. Sequence alignment of YjiC‐subbranch showed that the 66th and 77th amino acid residues, which were close to the catalytic cavity in molecular docking model, were conserved in five high‐active GTs (Q66 and P77) but changed in two low‐efficient GTs. Site‐directed residues swapping at the two positions in the two low‐active GTs (BssGT and BamGT) and the high‐active GT BsGT‐1 demonstrated that the two amino acid residues played an important role in the catalytic efficiency of epothilone glycosylation. This study highlights that the potent GTs for appointed compounds are phylogenetically grouped with conserved residues for the catalytic efficiency.

## Introduction

Glycosylation is a common modification mechanism of compounds, playing an important role in the biosynthesis of diverse glycoside natural products (Hancock *et al*., 2006). Glycosylation is able to alter compound properties in terms of water solubility, stability or bioactivity, which may be important in pharmaceutics (Kren and Martinkova, 2001; Thibodeaux *et al*., 2008; De Bruyn *et al*., 2015; Elshahawi *et al*., 2015). Glycosyltransferase (GT) is the enzyme responsible for transferring the glycosyl moiety from XDP‐activated glycosides to a variety of compounds. Based on the features of amino acid sequence, structure and glycosidic linkage, GTs are classified into 106 families (Lairson *et al*., 2008; Lombard *et al*., 2014). With the development of sequencing techniques, large numbers of GTs have been revealed in various microbial genomes, of which a few are known of their glycosylation specificity and efficiency (Hoffmeister *et al*., 2002; Blanchard and Thorson, 2006; Luzhetskyy and Bechthold, 2008). Exploring efficient GTs of these unexplored GT sequences are promising for the biosynthesis of glycoside compounds.

**Figure 1:**
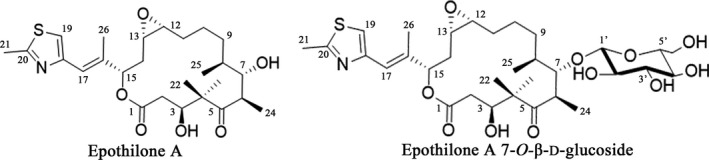
The structures of epothilone A and epothilone A 7‐*O*‐β‐d‐glucoside.

Epothilones are a kind of 16‐membered polyketides produced by the myxobacterium *Sorangium cellulosum* (Höfle *et al*., 1996). The compounds exhibit potent activities against multi‐drug resistant tumours (Kowalski *et al*., 1997; Giannakakou *et al*., 2000). Epothilone is able to bind to the β‐tubulin constituent to inhibit microtubule depolymerization, thus preventing mitosis and resulting in apoptosis of cell (Bollag *et al*., 1995). The inhibition effect is similar to that of Paclitaxel (Kowalski *et al*., 1997; Altmann *et al*., 2000), and Ixabepilone, a semi‐synthetic analogue of epothilone, has been approved by the Food and Drug Administration (FDA) for the treatment of cancer patients (Lee *et al*., 2001; Pronzato, 2008). However, the clinical usage of epothilones is limited by their significant neuro‐ and haematological toxicities (Cardoso *et al*., 2008; Chiorazzi *et al*., 2009). The undesired toxicity of epothilone may be decreased by glycosylation, because cancer cells are known to take in higher levels of glucose than normal cells, and thus the additional glycosyl group(s) could elevate the tumour selectivity of the epothilone glycoside(s) (Tanaka *et al*., 2011, 2013). Glycosylated epothilone compounds have been discovered in the fermentation broth of *Sorangium cellulosum* strain So0157‐2 with efficient cytotoxicity, for example, to human breast cancer cells (MDA‐MB‐435), human lung adenocarcinoma cells (A‐549) and mouse lympholeukemia cells (P‐388) (Li *et al*., 2010; Zhao *et al*., 2010). The GTs that are able to catalyse the glycosylation of epothilones have been less investigated, and YjiC, a uridine diphosphate‐dependent glycosyltransferase from *Bacillus licheniformis*, is so far the only GT reported with the glycosylation activity of epothilone A to epothilone A 7‐*O*‐β‐d‐glucoside (Parajuli *et al*., 2014) (Fig. 1). YjiC is a typical GT‐B fold enzyme possessing two separate domains (*N*‐terminal acceptor binding domain and the *C*‐terminal sugar donor binding domain) and exhibiting the promiscuous capacity to catalyse the glycosylation of other compounds, such as geldanamycin, resveratrol and daunomycin (Wu *et al*., 2012; Pandey *et al*., 2014a,2014b; Chu *et al*., 2016).

In this study, to search efficient GTs for epothilone glycosylation, we phylogenetically analysed the bacterial GT sequences available in CAZy database and revealed that YjiC was located at a subbranch of the Macrolide I branch in GT Family 1. We selected ten GTs from either the YjiC‐subbranch or non‐YjiC‐subbranches in the Macrolide I group to evaluate their glycosylation efficiencies on epothilone A. We found that five of the seven selected YjiC‐subbranch GTs were able to efficiently glycosylate epothilone A, and two were in low efficiency, while the three selected GTs from non‐YjiC‐subbranches in the Macrolide I branch had low or almost no epothilone glycosylation activity. Sequence alignment showed that four amino acid positions are conserved in the five highly efficient GTs but changed in the two low‐efficient YjiC‐subbranch GTs. We performed site‐directed mutagenesis on these four residues and determined that the two residues (Q66 and P77) played an important role in the catalytic efficiency, while the other two residues had low effects on epothilone glycosylation. This study demonstrates that phylogenetic approach is an efficient means for screening GTs for appointed compounds.

## Results and Discussion

### Phylogenetic analysis and selection of GTs for epothilone glycosylation

**Figure 2:**
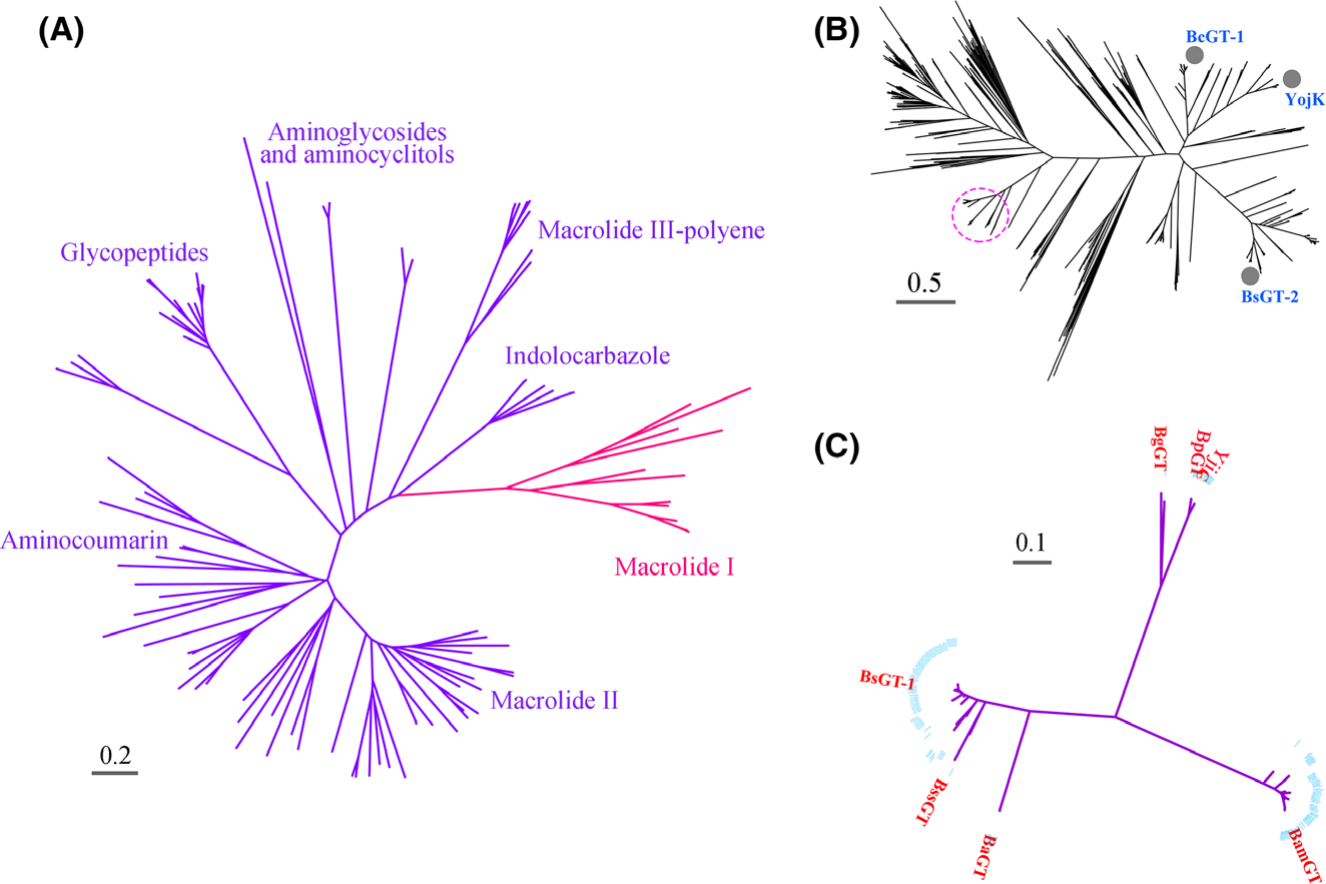
Phylogenetic analysis of GTs with YjiC as the reference. A. A phylogenetic tree constructed with representatives of the available 8261 GT sequences from GT Family 1 in the Cazy database. YjiC is located in the Macrolide I group. B. A phylogenetic analysis of the 1481 available members in the Macrolide I group. YjiC was in the subbranch containing 161 GT sequences (in a red cycle). The three marked sequences were selected as the non‐YjiC‐subbranch GTs for the glycosylation assays on epothilone A. C. A phylogenetic tree showing that the 161 GT sequences in the YjiC‐subbranch were still diverse. The YjiC‐subbranch divided into three major small groups. The seven marked GT sequences were selected as the YjiC‐subbranch GTs for the epothilone glycosylation assays.

YjiC belongs to the Family 1 GTs. In order to explore the epothilone GTs, we phylogenetically analysed 8261 bacterial GT sequences from GT Family 1 available in the Cazy database. Figure 2A is a phylogenetic tree constructed with representatives in different branches, and some branches in the phylogenetic tree were named according to the elucidated functions of GTs (Liu *et al*., 2016). The YjiC sequence was located at the Macrolide I branch. Totally, there were 1481 GT sequences in the Macrolide I branch, and some of which had been previously reported to play glycosylation functions in the biosynthesis of different compounds. For example, OleD is a *Streptomyces* GT functioning in the glycosylation of oleandomycin (Bolam *et al*., 2007), YojK and BcGT‐1 from *Bacillus* involve in the biosyntheses of ginsenosides (Luo *et al*., 2015) and flavonoids (Hyung Ko *et al*., 2006) respectively. Notably, many reported GTs in the Macrolide I branch are capable of catalysing the glycosylation in a regioselective manner (Zhou *et al*., 2013; Pandey *et al*., 2014a,2014b). The Macrolide I GT sequences are greatly diverse, including many small subbranches. Figure 2B is a phylogenetic tree constructed with the 1481 GTs. YjiC was in a subbranch (circled on the tree) containing 161 members, which are all from *Bacillus* spp. (Table S1). In addition to YjiC, some other GTs in this subbranch, for example BsGT‐1 (Dai *et al*., 2017), have also been reported the glycosylation activities, but most sequences have not yet been investigated.

We inferred that GTs in the YjiC‐subbranch, or even the Macrolide I branch, potentially have the glycosylation capacity of epothilone. The 161 GT sequences in the YjiC‐subbranch were further divided into three major groups (Fig. 2C). We selected seven GTs from different groups (marked in the tree) in the YjiC‐subbranch, including YjiC, to determine their glycosylation activities on epothilone. In addition, we also chose three GTs from non‐YjiC‐subbranches in the Macrolide I group, that is *Bacillus*‐derived YojK, BcGT‐1 and BsGT‐2 (marked blue in Fig. 2B). Multi‐sequence alignment indicated that, of the selected GTs, BpGT is phylogenetically closest to YjiC (the sequence identity is 94.4%), while BsGT‐2 is farthest to YjiC with the sequence identity of 27.4% (Table S2). Table 1 summarizes the information of these ten GTs.

**Table 1:** Information of the glycosyltransferases selected for epothilone A glycosylation

Enzyme	Genbank number	Number of amino acids	Molecular weight (kDa)	Isoelectric point	Sources of strains	Phylogenetic sites	Structure modelling	Compare to OleD	
							Estimated TM‐score	RMSD	Identity
YjiC	AAU40842	396	44.66	5.13	*Bacillus licheniformis* DSM 13	YjiC‐subbranch	0.92±0.06	3.42	0.312
BsGT‐1	CUB50191	392	43.97	4.85	*B. subtilis* JRS11	YjiC‐subbranch	0.80±0.09	2.64	0.299
BaGT	ADP31706	393	44.19	4.78	*B. atrophaeus* 1942	YjiC‐subbranch	0.90±0.06	3.40	0.269
BgGT	SCA85980	394	45.07	5.39	*B. glycinifermentans* BGLY 2157	YjiC‐subbranch	0.87±0.07	3.34	0.295
BpGT	ARA85718	396	44.63	5.04	*B. paralicheniformis* MDJK30	YjiC‐subbranch	0.85±0.08	3.31	0.298
BssGT	AMA51908	392	44.09	5.07	*B. subtilis* subsp. inaquosorum DE111	YjiC‐subbranch	0.82±0.08	3.47	0.282
BamGT	AKD21753	394	44.34	5.00	*B. amyloliquefaciens* L‐S60	YjiC‐subbranch	0.86±0.07	3.36	0.285
YojK	NP389824	405	45.60	5.09	*B. subtilis* str. 168	Non‐YjiC‐subbranch	0.90±0.06	3.46	0.230
BcGT‐1	AAS41089	400	45.55	5.34	*B. cereus* ATCC 10987	Non‐YjiC‐subbranch	0.81±0.09	0.66	0.246
BsGT‐2	WP003234124	395	44.55	5.31	*B. subtilis* JRS11	Non‐YjiC‐subbranch	0.86±0.07	3.31	0.170

BaGT, *Bacillus atrophaeus* glycosyltransferase; BamGT, *Bacillus amyloliquefaciens* glycosyltransferase; BcGT‐1, *Bacillus cereus* glycosyltransferase‐1; BgGT, *Bacillus glycinifermentans* glycosyltransferase; BpGT, *Bacillus paralicheniformis* glycosyltransferase; BsGT‐1, *Bacillus subtilis* glycosyltransferase‐1; BsGT‐2, *Bacillus subtilis* glycosyltransferase‐2; BssGT, *Bacillus subtilis subsp* glycosyltransferase.

### *In vitro* epothilone glycosylation activities of purified GTs

**Figure 3:**
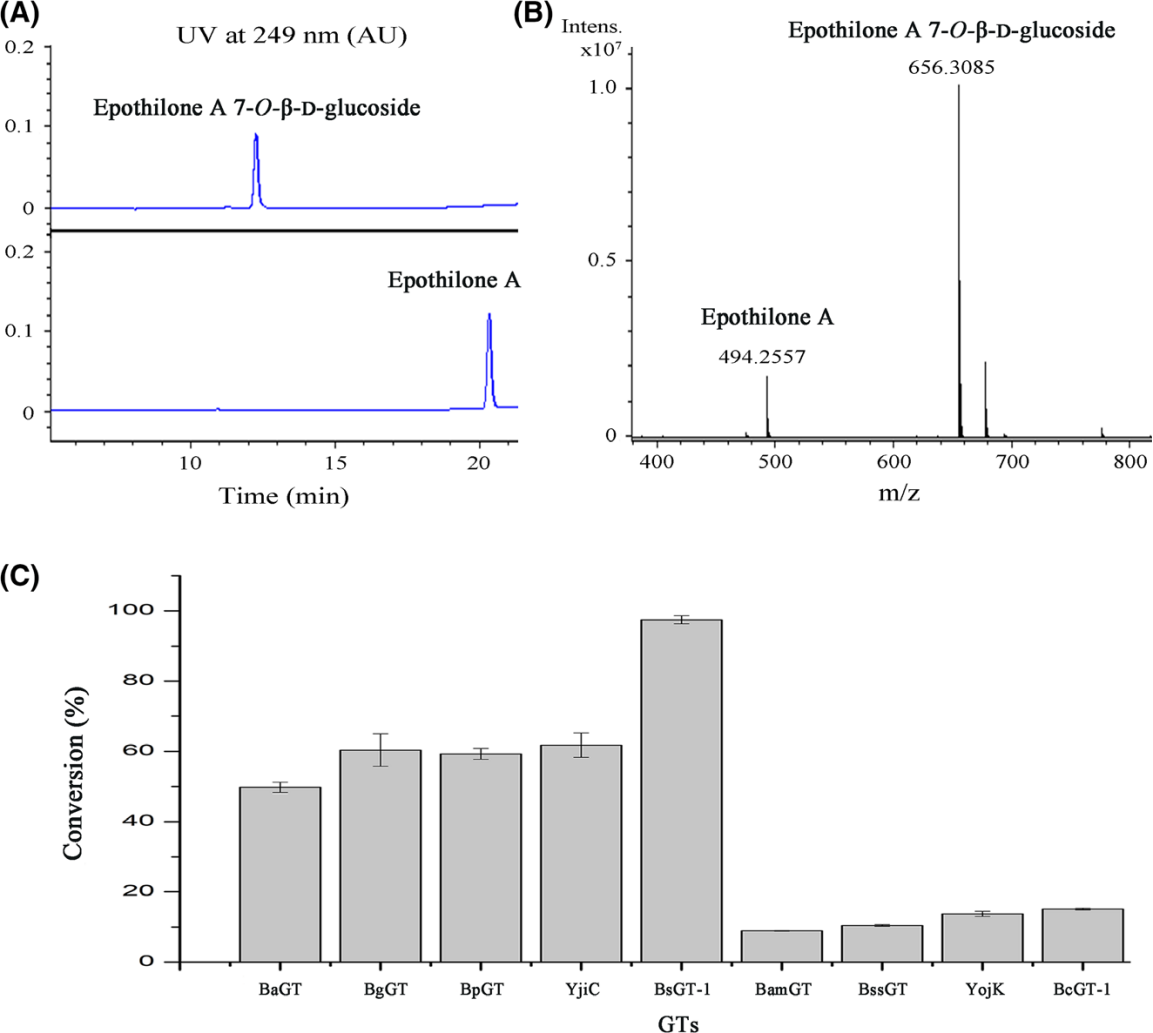
Conversion from epothilone A to epothilone A glucoside by GTs. The HPLC (A) and the MS (B) detection of epothilone A and epothilone A glucoside. C. The conversion efficiencies by different GTs after 2 h of incubation.

We constructed each of the ten encoding genes into the pET28a plasmid and expressed the ten GTs separately in *E. coli* BL21 (DE3). All the GT proteins were expressed solubly (Fig. S1), which were purified (the optimal imidazole concentration for purification and SDS‐PAGE verification of the purified proteins are demonstrated in Fig. S2). The following *in vitro* glycosylation assays were performed with the purified proteins, and the glycosylated products were determined with the UPLC‐PDA and HR‐QTOF ESI‐MS techniques. Consistent with the previous report (Parajuli *et al*., 2014), YjiC was able to glycosylate epothilone A into epothilone A glucoside. Figure 3A and B demonstrates the HPLC and the MS results of epothilone A glucoside produced by YjiC. Similarly, eight of the other nine *Bacillus*‐derived GT proteins exhibited the glycosylation abilities on epothilone A, except BsGT‐2. The glycosylated products from different GTs all had the same HPLC‐retention time, and no epothilone A glucoside peak was observed in the reaction with BsGT‐2 (the HPLC results are provided in Fig. S3).

To verify the glycosylation, the product produced by BsGT‐1 was purified. The experimental 1D NMR data assignment of the compound is summarized in Table S3, which is consistent with the previous results (Parajuli *et al*., 2014). The glycosylation at C‐7 was determined by the key HMBC correlations from H‐7 to C‐1′, and from H‐1′ to C‐7. The *O*‐β‐linkage between aglycone and the d‐glucose was revealed by the big coupling constant (*J*_1′, 2′_, 7.8 Hz) between the anomeric proton and H‐2^′^, as well as the chemical shift of anomeric proton H‐1^′^ at *δ*_H_ 4.46 ppm. The proofs for NMR identification of epothilone A 7‐*O*‐β‐d‐glucoside are provided in Fig. S4–S8.

Among the seven YjiC‐subbranch GTs, five were efficient in the glycosylation of epothilone A. BsGT‐1 almost completely conversed epothilone A into epothilone A glucoside after 2 h of incubation (97.5%, calculated from the HPLC peaks), whereas the conversion efficiencies of BaGT, BgGT and BpGT were 49.8%, 60.8% and 59.3%, respectively, which were similar to that of YjiC (61.8%). The conversion efficiencies by the other two YjiC‐subbranch GTs, BssGT and BamGT were 10.50% and 8.90% respectively. Comparatively, of the three non‐YjiC‐subbranch GTs, while BsGT‐2 showed no HPLC peak of epothilone A, YojK and BcGT‐1 had conversion efficiencies of 13.7% and 15.1% after 2 h of incubation respectively. Figure 3C exhibits the conversion efficiencies by the nine active GTs after 2 h of incubation.

### Enzymatic and kinetic characteristics of the epothilone GTs

**Figure 4:**
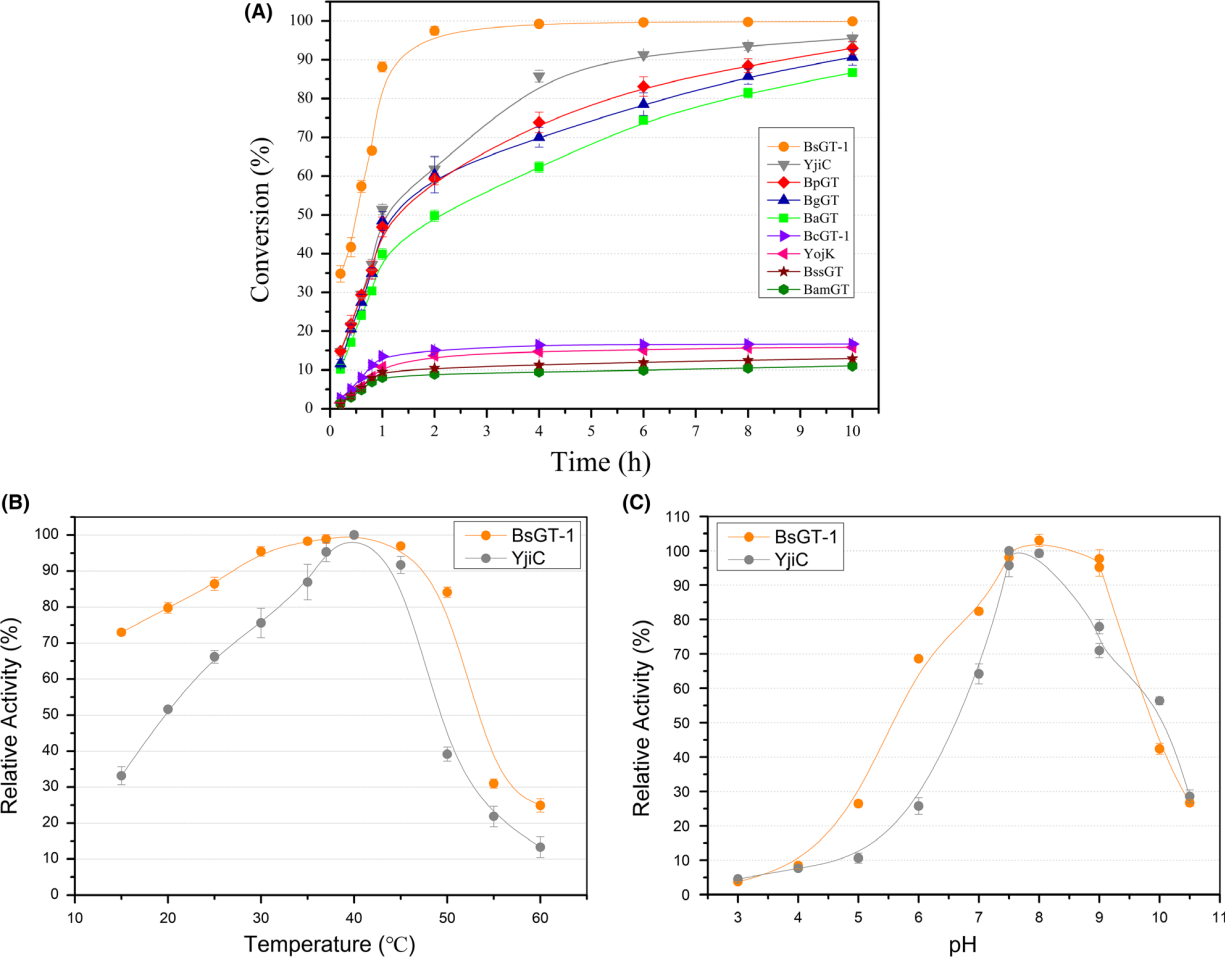
Enzymatic characteristics of epothilone GTs. A. The conversion curves of nine GTs. B. Comparison of the optimal temperature for the glycosylation of epothilone A by BsGT‐1 and YjiC (Setting relative activity at 40°C as 100% of each enzyme). C. Comparison of the optimal pH value for the glycosylation of epothilone A by BsGT‐1 and YjiC (Setting the relative activity in Tris–HCl buffer, pH 7.5, as 100% of each enzyme). The incubation time for the reaction was 2 h.

To evaluate the conversion reactions of the GTs in detail, we composed a conversion curve for each of the nine GTs (Fig. 4A). While BsGT‐1 achieved almost complete conversion in short time, the conversion by BaGT, BgGT, BpGT or YjiC increased continuously and reached approximately 90% or higher after 10 h of incubation. BssGT and BamGT almost achieved the equilibrium at low levels before 2 h, and additional incubation did not further change the conversion, which is consistent with the results of the two low‐active GTs from non‐YjiC‐subbranches. Notably, the LC‐MS detection indicated that epothilone A glucoside could be produced in the reaction mixture with BsGT‐2, but the conversion efficiency was only 0.03% after 10 h of incubation. Thus, all the ten assayed *Bacillus*‐derived GTs were able to glycosylate epothilone A, but in distinct efficiencies. Obviously, the GTs from the YjiC‐subbranch are more efficient than the non‐YjiC‐subbranch GTs for epothilone glycosylation. The capacity for epothilone glycosylation is related to the sequence phylogeny, which is consistent with the conclusion that the GT members with close genetic relationships normally possess the similar substrate specificity (Liu *et al*., 2016).

Among the assayed GTs, BsGT‐1 exhibited excellent glycosylation activity on epothilone not only in the conversion efficiency but also in the rate. This enzyme was previously reported to be capable of catalysing the glycosylation reactions promiscuously for a variety of natural and unnatural products, including flavonoids, phenyl ketones, curcuminoid, lignins, zingerone, triterpenes, stilbene, anthraquinone and aromatic model aglycons with nucleophilic groups of −OH, −NH_2_ or −SH (Dai *et al*., 2017). BsGT‐1 possesses 57% identity of its amino acid sequence with that of YjiC, but the glycosylation efficiency by BsGT‐1 was greatly higher than that by YjiC. We assayed the optimal reaction temperatures and pH values of BsGT‐1 and YjiC to compare their enzymatic characteristics for epothilone A glycosylation. These two enzymes had similar optimal temperatures and acid‐alkali tolerances, yet to different extents. YjiC retained more than 80% activity in the range of 30 and 45°C. Comparatively, BsGT‐1 showed a wider temperature tolerance, having more than 80% activity within the range of 20 and 50°C (Fig. 4B). The two GTs both decreased the enzymatic activity rapidly with the temperature higher than 50°C. Similarly, the optimum pH value (relative activity > 80%) was at 7.0–9.0 for YjiC, and 6.5–9.5 for BsGT‐1 (Fig. 4C). Thus, compared with YjiC, BsGT‐1 also possessed wider temperature and pH tolerance.

Furthermore, kinetic characteristics of the seven selected epothilone GTs were assayed. As summarized in Table 2, the Km values for the glycosylation of the highly active GTs, BsGT‐1, YjiC, BaGT, BgGT and BpGT on epothilone A were 57.79, 94.58, 113.66, 132.37 and 89.79 μM, respectively, while the Km values for the low‐active GTs, BssGT, BamGT, YojK and BcGT‐1 were all higher than 1000 μM. The *k*_cat_/*K*_m_ values of the highly active GTs were also significantly higher than that of the low‐active GTs (Table 2, Fig. S9). For example, the *k*_cat_/*K*_m_ of BsGT‐1 was 305.59 min^−1^ mM^−1^, which is about 400‐fold higher than that of BssGT (0.72 min^−1^ mM^−1^). The above results showed that, among the assayed GTs, BsGT‐1 is the most effective biocatalyst for epothilone glycosylation.

**Table 2:** Kinetic parameters of epothilone GTs on epothilone A

Epothilone GTs	Acceptor substrate (epothilone A)		
	*K*_m_ (μM)	*K*_cat_ (min^−1^)	*K*_cat_/*K*_m_ (min^−1^ mM^−1^)
BsGT‐1	57.79 ± 4.98	17.66 ± 0.34	305.59
YjiC	94.58 ± 2.95	11.53 ± 0.19	121.91
BaGT	113.66 ± 10.55	6.75 ± 0.25	59.39
BgGT	132.37 ± 10.91	10.31 ± 0.40	77.89
BpGT	89.79 ± 9.02	9.70 ± 0.30	108.03
BssGT	1585.26 ± 116.74	1.14 ± 0.05	0.72
BamGT	2245.74 ± 311.96	1.09 ± 0.070	0.50
YojK	1720.77 ± 161.35	1.23 ± 0.07	0.71
BcGT‐1	1196.12 ± 214.98	1.07 ± 0.07	0.89
BsGT‐2	ND	NA	–

NA, no activity detected; ND, not determined.

### Sequence and structural analyses of YjiC‐subbranch GTs

**Figure 5:**
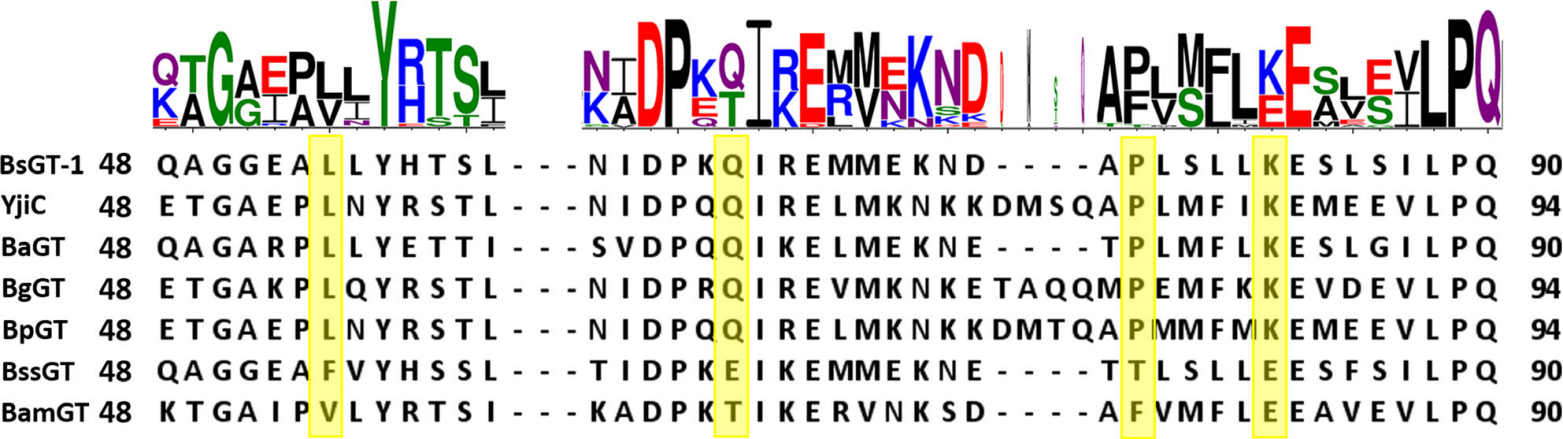
Multiple sequence alignment of the ten assayed GTs with OleD as reference. The positions of L54, Q66, P77 and K82 (based on BsGT‐1) are highlighted yellow in five high‐active GTs and two low‐active GTs. Sequence consensus of the above four positions in all the 161 GTs from YjiC‐subbranch is visualized by WebLogo.

The selected seven YjiC‐subbranch GTs showed distinct glycosylation activities, but their amino acid sequence identities were higher than 53.3%, and the sequence identity between BsGT‐1 and BssGT even reached 88% (Table S2). In order to investigate the difference of glycosylation efficiency, we performed sequence alignment of the seven selected YjiC‐subbranch GTs, which showed that four amino acid residues were conserved in the five efficient GTs (L54, Q66, P77 and K82, numbered based on BsGT‐1), but changed in the low‐active GTs (Fig. 5). Notably, the four amino acid residues were also varied in the non‐YjiC‐subbranch GTs (Fig. S10). We compared sequences of all the 161 members in the YjiC‐subbranch GTs (Fig. S11), which showed that the amino acid residues at each of the four positions were mainly occupied by two kinds of amino acid residues, that is L54 or V54, Q66 or T66, P77 or F77, and K82 or E82.

**Figure 6:**
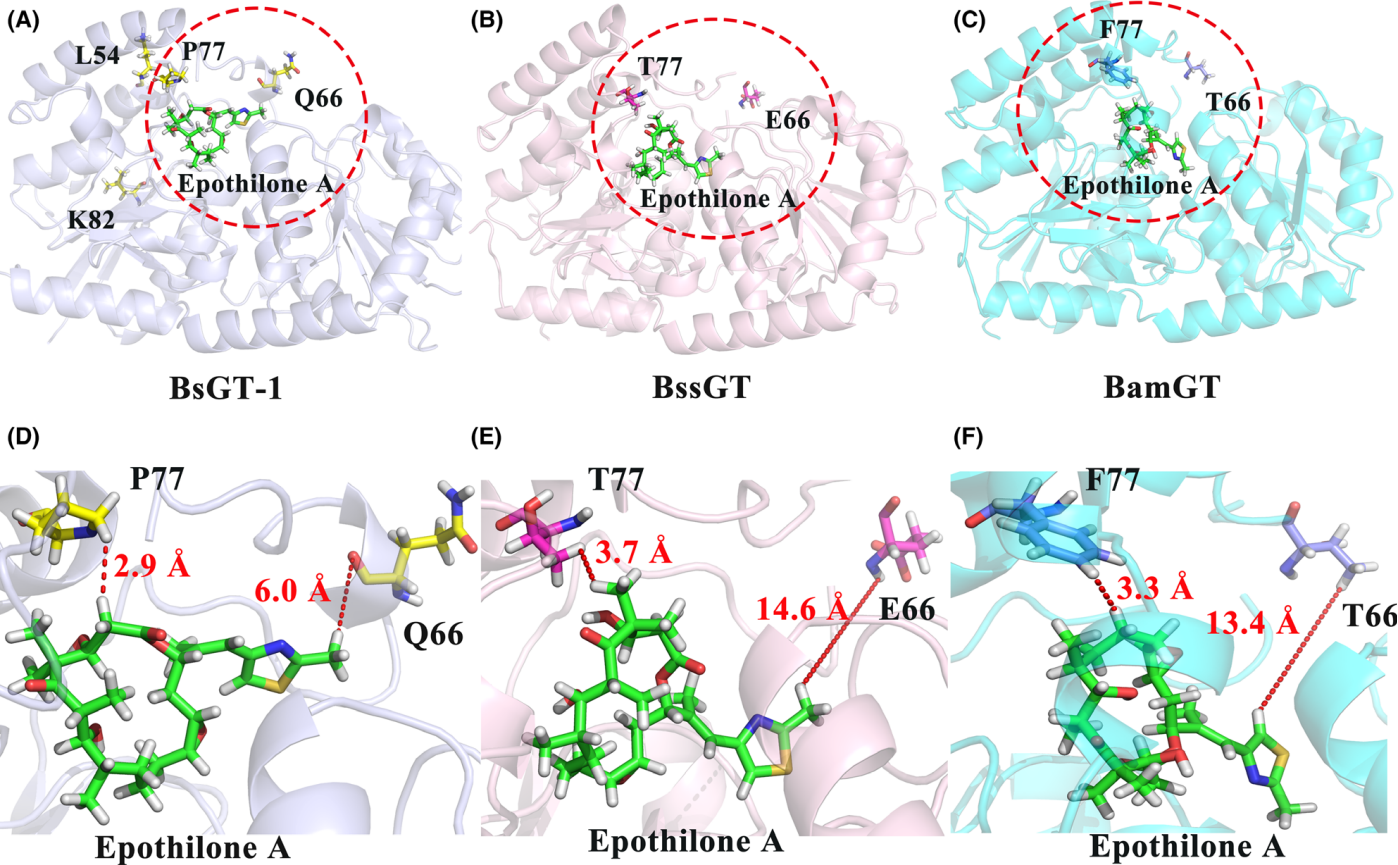
Structure modelling and docking of the GTs. A–C. The epothilone A (green) was docked into the hydrophobic pockets of BsGT‐1 (light grey), BssGT (light pink) and BamGT (light blue) respectively. D. An amplified perspective of the positional relation between epothilone A (green) with two influential residues Q66 and P77 (yellow) of BsGT‐1. E. The positional relation between substrate (green) with E66 and T77 (pale red) of BssGT. F. The positional relationship between substrate (green) and two residues (T66, F77, blue) of BamGT.

In the Macrolide I group, the crystal structure of OleD has been elucidated (PDB 2YIA) (Bolam *et al*., 2007). To investigate the sequence and structural characteristics, we built three‐dimensional structures of epothilone GTs using the threading method with OleD as the reference (Zhang, 2008). After modelling, the structures were refined at the atomic level by the fragment‐guided molecular dynamics (FG‐MD) simulations (Zhang *et al*., 2011). The TM‐scores, the values from the algorithm for sequence‐order independent protein structure comparison (Zhang and Skolnick, 2005), were higher than 0.8 for the seven YjiC‐subbranch GTs, which is greatly higher than the threshold of 0.5, suggesting the same folding with acceptable modelling accuracy of the two compared proteins. Information of the structure models is provided in Table 1. The three‐dimensional (3D) structures (Fig. 6) showed that BsGT‐1, BssGT and BamGT all have the typical GT‐B fold, containing two Rossmann‐like domains (*N*‐terminal acceptor binding domain and the C‐terminal sugar donor binding domain). The two domains, which form a fuzzy catalytic cavity, are able to move globally to bind substrate specifically in different types and degrees (Vrielink *et al*., 1994; Chang *et al*., 2011). Although the BsGT‐1 and BssGT showed a high degree of amino acid sequence identity (88%, Table S2) and structural similarity (Fig. 6A and B), they displayed distinct catalytic activities. We docked epothilone A into the hydrophobic pocket, which showed that the 66th and the 77th amino acids of BsGT‐1, BssGT and BamGT were all near the catalytic cavity, while the 54th and the 82nd amino acids were rather far from the cavity (Fig. 6A).

### Site‐directed amino acid swapping of epothilone GTs

**Figure 7:**
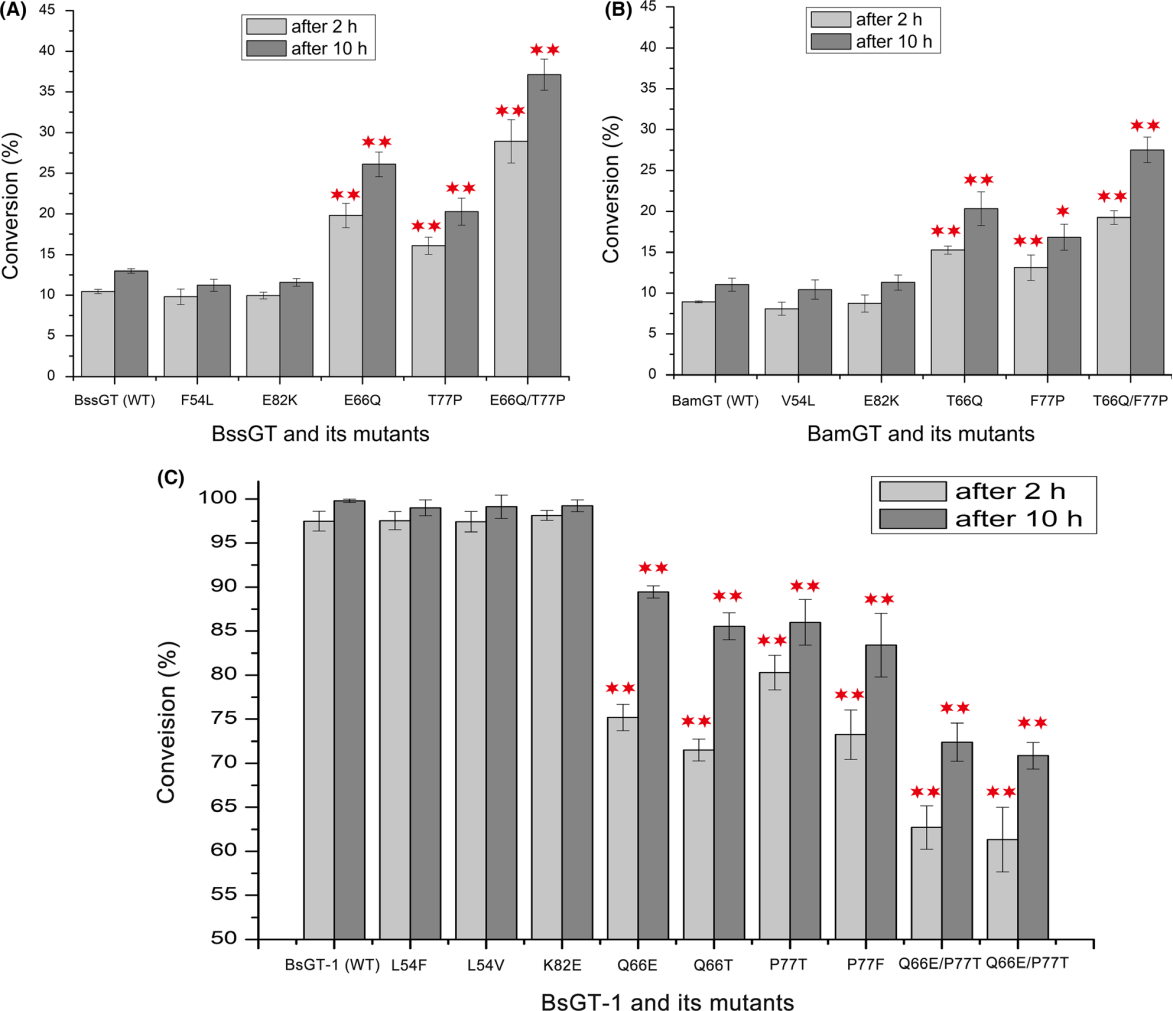
Glycosylation conversion rates of GTs and their mutants after 2 h and 10 h (**P < 0.01 and *P < 0.05). A‐B. Glycosylation conversion rates of two low active GTs (BssGT and BamGT) and their swapping mutants of the four sites (54th, 82th, 66th and 77th). C. Glycosylation conversion rates of the high active GT (BsGT‐1) and its corresponding swapping mutants.

To determine whether the four conserved amino acid residues were important for glycosylation efficiency, we performed site‐directed amino acid swapping at the four positions of BsGT‐1, BssGT and BamGT, respectively. The F54L or E82K swapping of BssGT, or the V54L or E82K of BamGT did not improve epothilone glycosylation activities of the enzymes (Fig. 7A and B). However, E66Q or T77P of BssGT increased the activity significantly, and the conversion rates by the mutants reached 26.09% and 20.27% after 10 h incubation respectively. Further, double swapping of the two amino acids (E66Q/T77P) had an approximate three‐fold increase of the enzymatic activity, and the conversion rate reached 37.11% after 10 h incubation. The T66Q, F77P and T66Q/F77P swapping in BamGT exhibited similar results as that in the BssGT mutations (Fig. 7A and B). We also performed swapping in BsGT‐1 to confirm the functions of amino acids at the four positions. Q66E and P77T (mutation to BssGT) caused significant decreases of glycosylation efficiency, and the conversion rates were 89.45% and 85.99% after 10 h incubation respectively. Double swapping of the two residues led to the conversion rate of the mutant (Q66E/P77T) to 72.4%. The Q66T, P77F and Q66T/P77F swapping (mutation to BamGT) exhibited similar conversion rates. Mutations at the L54 and K82 residues in BsGT‐1 had no significant effects on the glycosylation efficiency of epothilone A (Fig. 7C). In the docking models, the distance between Q66 (BsGT‐1) and epothilone A was 6.0 Å, while the corresponding residues in BssGT (E66) and BamGT (T66) were rather far away from substrate (> 10 Å), which probably affected the glycosylation efficiency of the three GTs. Comparatively, the 77th amino acids of the three GTs were all close to the substrate in the docking models with small differences, *that is* 2.9 Å (P77 of BsGT‐1), 3.7 Å (T77 of BssGT) and 3.3 Å (F77 of BamGT) respectively (Fig. 6D–F). In BsGT‐1, the proline amino acid at the 77th position probably limited the conformational flexibility in the cavity of the protein, providing a mild and stable hydrophobic environment. In contrast, the polar amino acid threonine (BssGT) or the amino acid with large side‐chain phenylalanine (BamGT) at the 77th position might affect the rigidity of the catalytic cavity, driving the substrate away from the catalytic sites. Swapping the amino acid at the 66th or 77th position in highly active BsGT‐1 or in low‐active BssGT or BamGT probably induced conformational changes, but not dramatically, since the glycosylation activities were only partially decreased or increased. Conclusively, Q66 and P77 of the YjiC‐subbranch GTs had an important role in the epothilone glycosylation.

## Conclusion

YjiC locates in the Macrolide I group of Family 1 of glycosyltransferases. Our studies showed that the GTs in the Macrolide I group have potential glycosylation activities on epothilone, and the YjiC‐subbranch GTs often contain the GTs with high efficiency for the epothilone glycosylation. Similar structures of the YjiC‐subbranch GTs suggest their glycosylation mechanisms on epothilone are also similar. We demonstrate that Q66 and P77 play an important role for epothilone glycosylation efficiency. Although swapping of the two amino acid residues increased enzymatic efficiency of the low‐active epothilone GTs, the mutants did not display the potent glycosylation activity as BsGT‐1. It is worthwhile to explore more details about the catalytic mechanism of GTs by sequence motif analysis, mutation and crystallization.

## Experimental procedures

### Chemicals and reagents

UDP‐d‐glucose (UDPG) was purchased from Sigma‐Aldrich (St. Louis, MO, USA). Acetonitrile and methanol were obtained from Thermo Fisher Scientific (Waltham, MA, USA). All chemicals and reagents are of analytical grade.

### Bacterial strains, plasmids, media and culture conditions

*Escherichia coli* Top 10 competent cells (Invitrogen, Carlsbad, CA, USA) were used as the host for the construction of recombinant vectors. *E. coli* BL21 (DE3) (Invitrogen) harbouring the recombinant pET28a vector (Novagen, Madison, WI, USA) was used for the protein expression. The bacteria were cultivated at 37°C in Luria–Bertani (LB) medium containing 1.0% tryptone (Oxoid, Hampshire, UK), 0.5% yeast extract (Oxoid, Hampshire, UK) and 0.5% NaCl. The liquid cultures were shaken at 200 r/min in a shaking incubator.

### The GTs encoding genes

The genes encoding YjiC (AAU40842), YojK (NP389824), BcGT‐1 (AAS41089), BsGT‐1 (CUB50191), BsGT‐2 (WP003234124), BaGT (ADP31706), BssGT (AMA51908), BamGT (AKD21753), BgGT (SCA85980) and BpGT (ARA85718) were retrieved from the CAZy database and synthesized in VoyaGene (VoyaGene Inc, Hangzhou, China). These gene sequences were, respectively, constructed into the pET28a vector, and the recombinant plasmids were transformed into *E. coli* BL21 (DE3) for the protein expression by using the previously described protocol (Yue *et al*., 2017).

### Expression and purification of glycosyltransferases

Transformants were grown in 2 ml LB medium supplemented with kanamycin at the final concentration of 40 μg ml^−1^ for 12 h, and then the 2 ml cultures were transferred into a 300 ml flask containing 100 ml LB medium supplemented with 40 μg ml^−1^ kanamycin for large‐scale cultivation. When the optical density at 600 nm (OD_600_) of the cultures reached 0.6, isopropyl‐β‐d‐1‐thiogalactopyranoside (IPTG) was added at the final concentration of 0.1 mM. The cultures were shaken at 16°C for additional 24 h. Then, the cell pellets were harvested by centrifugation at 8000 r.p.m. and 4°C for 5 min, washed twice with a Tris–HCl buffer (50 mM Tris–HCl, 10% glycerol, pH 7.5) and resuspended in 10 ml of the same buffer. The cells were sonicated, and the supernatant was collected by centrifugation at 14 000 r.p.m. and 4°C for 30 min. The protein expressions were analysed by the 12% sodium dodecyl sulphate polyacrylamide gel electrophoresis (SDS‐PAGE).

The GT proteins were purified using a His‐TALON metal nickel affinity resin (Takara Bio, Shiga, Japan). The supernatants were added to a pretreated nickel affinity resin and incubated at 4°C for 12 h. The resin‐bound proteins were eluted with a Tris–HCl buffer (50 mM Tris–HCl and 10% glycerol, pH 7.5) containing different concentrations of imidazole (20 mM, 50 mM, 100 mM, 150 mM and 250 mM), and the elutes were analysed by SDS‐PAGE. Imidazole was removed from the protein solution by using the 10 kD ultrafiltration tubes (Paul, USA), and the concentrated protein samples were stored at −20°C for purified enzymes assays.

### *In vitro* glycosylation reactions of GTs and their kinetic parameters

The purified GTs were subject to *in vitro* glycosylation reactions. The reaction was performed in a mixture (50 μl) containing the final concentrations of 50 mM Tris–HCl buffer, 10 mM MgCl_2_, 3 mM UDPG, 0.5 mM epothilone A (dissolved in DMSO) and 400 μg ml^−1^ of each protein. To determine the conversion changes with the incubation time, the reactions were sampled at different time points (0.2, 0.4, 0.6, 0.8, 1, 2, 4, 6, 8 and 10 h).

To investigate effects of temperature on glycosylation, the reaction mixtures in Tris–HCl buffer (50 mM Tris–HCl, 10% glycerol, pH 7.5) were incubated at different temperatures ranging from 15 to 60°C for 2 h. Similarly, the reactions were performed at 37°C for 2 h with different pH values ranging from 3.0 to 10.5 (3.0–7.5 in McIlvaine buffer; 7.5–9.0 in Tris–HCl buffer and 9.0–10.5 in glycine‐NaOH buffer) to determine the optimal pH for glycosylation.

Kinetic analysis of the GTs for the glycosylation of epothilone A was performed in the reaction mixture (50 μl) containing 1 μg purified protein, 50 mM Tris–HCl buffer (50 mM Tris–HCl, pH 7.5), 10 mM MgCl_2_, 10 mM UDPG and different concentrations of epothilone A (Fig. S9). The mixtures were incubated at 37°C for 10 min. All tests were performed in three replicates. The kinetic values were determined by fitting the Michaelis–Menten curve to the data using the nonlinear regression method.

All the above reactions were terminated with a triple volume of methanol. After centrifugation at 14 000 r.p.m. for 30 min to remove protein precipitates, the reaction mixtures were analysed with UPLC‐PDA and HR‐QTOF ESI‐MS.

### UPLC‐PDA and HR‐QTOF ESI‐MS assays

The samples were subjected to the UPLC‐PDA and HR‐QTOF ESI‐MS instruments equipped with the C_18_ column (Thermo Fisher Scientific, C_18_, 250 mm×4.6 mm, 5 μm). Products were detected by the UV absorbance at 249 nm. The binary mobile phase included solvent A (HPLC‐grade purity water, 0.1% formic acid) and solvent B (HPLC‐grade purity acetonitrile, 0.1% formic acid) at a flow rate of 1 ml min^−1^ for 40 min. The acetonitrile concentrations were changed as follows: 30–35% (0–15 min), 35–75% (15–25 min), 75–90% (25–30 min) and 90–5% (35–40 min). 1D and 2D NMR spectra were recorded on a Bruker Avance III‐600 NMR spectrometer (Bruker, Billerica, MA, USA) equipped with CryoprobeTM.

### Site‐directed mutagenesis of epothilone GTs

Fast Mutagenesis Kit V2 (Vazyme Biotech Co., Ltd, Nanjing, China) was used for the site‐directed mutagenesis of epothilone GTs. After sequencing confirmation, the mutants were subject to the protein expression and purification as the above described, and the purified mutant proteins were used to evaluate the epothilone A glycosylation efficiencies.

### Bioinformatics analyses

The ExPASy compute pI/Mw tool was used to predict the theoretical molecular weight and isoelectric points of GTs (Wilkins *et al*., 1999). The GTs sequences were aligned by using MAFFT (version 7) (Katoh *et al*., 2013). FastTree was used to construct approximately maximum‐likelihood phylogenetic trees (Price *et al*., 2009). To infer a tree for a protein alignment with the JTT+CAT model (Jones *et al*., 1992) and to quickly estimate the reliability of each split in the tree, FastTree computes local support values with the Shimodaira–Hasegawa test (Price *et al*., 2009). All the GT amino acid sequences employed in the bioinformatics analysis were retrieved from the CAZy database (Letunic and Bork, 2011).

The I‐TASSER method was used to predict the protein structures. I‐TASSER is a hierarchical method for protein structure prediction (Zhang, 2008). The structural templates were first identified from the PDB databases by the multiple‐threading programme LOMETS. Then, the full‐length models were constructed by the iterative template fragment assembly simulation method. TM‐align was used for independent protein structures comparisons (Zhang and Skolnick, 2005). Docking experiments were performed using Swiss‐dock (Grosdidier *et al*., 2011).

## Conflict of interest

None declared.

## Author contributions

PZ and YZL designed researches; PZ, QC, YG, XJY performed researches; PZ, ZZ, ZFL, YYL, DHS, YMZ, CSW and YZL analysed data; PZ, CSW and YZL wrote the paper. YZL provided funds for the project.

## Supporting information


**Figure S1**. SDS‐PAGE detection of the expression of different GTs. Each of the heterologously expressed GTs contains a His tag. All of the GTs were soluble. M is the protein molecular weight marker, Lane 1, the control of pET 28a vector; Lanes 2–11, the *E. coli* lysate before induction; Lanes 12–21, the *E. coli* lysate after induction; Lanes 22–31, the soluble supernatants after induction.
**Figure S2**. SDS‐PAGE detection of the purification of YjiC and YojK proteins. (A). The purification with different concentrations of imidazole. Lanes 1–3, the purification of YjiC with 50 mM imidazole; Lanes 4–7, the purification of YjiC with 100 mM imidazole. Lanes 8–10, the purification of YojK with 50 mM imidazole; Lanes 11–14, the purification of YojK with 100 mM imidazole. (B). The purified YjiC, BsGT‐1, YojK and BsGT‐1. M is the protein molecular weight marker.
**Figure S3**. HPLC detection of the glycosylation products from epothilone A by purified GTs. The control is the standard of epothilone A. The reactions were performed for 2 h of incubation time.
**Figure S4.** ^1^H NMR (600 MHz) of epothilone A 7‐O‐β‐d glucoside in CD_3_OD.
**Figure S5.** ^13^C NMR (150 MHz) of epothilone A 7‐O‐β‐d glucoside in CD_3_OD.
**Figure S6.** COSY of epothilone A 7‐O‐β‐d glucoside in CD_3_OD.
**Figure S7.** HSQC of epothilone A 7‐O‐β‐d glucoside in CD_3_OD.
**Figure S8.** HMBC of epothilone A 7‐O‐β‐d glucoside in CD_3_OD.
**Figure S9**. **(A)** Kinetic parameters and curves analysis of high‐active GTs‐catalyzed reactions. Determination of kinetic parameters for epothilone A with saturated UDP‐d‐glucose (10 mM): epothilone A was set as different concentrations from 10–400 μM. Enzyme assays were performed in 50 mM Tris–HCl buffer (pH 7.5) containing 20 μg ml^−1^ GTs and 10 mM MgCl_2_ at 37°C for 10 min in triplicate. **(B)** Kinetic parameters and curves analysis of low‐active GTs‐catalyzed reactions. Determination of kinetic parameters for epothilone A with saturated UDP‐d‐glucose (10 mM): epothilone A was set as different concentrations from 100–4000 μM. Enzyme assays were performed in 50 mM Tris–HCl buffer (pH 7.5) containing 20 μg ml^−1^ GTs and 10 mM MgCl_2_ at 37°C for 10 min in triplicate.
**Figure S10**. Multiple sequence alignment of the ten assayed GTs with OleD as reference. The sites of L54, Q66, P77 and K82 (based on BsGT‐1) are highlighted yellow in five high active GTs and two low active GTs.
**Figure S11**. The WebLogo shows the amino acid residues comparison of the 161 GT members in the YjiC‐subbranch. The four amino acid positions (L54, Q66, P77 and K82, based on BsGT‐1) were marked with red boxes and their actual locations (based on BsGT‐1) were numbered.
**Table S1.** 161 numbers in the YjiC‐subbranch GTs.
**Table S2.** Amino acid identity among the ten selected GTs.
**Table S3.** 1D NMR (600 MHz, CD_3_OD) data for epothilone 7‐*O*‐β‐d glucoside.
