# Supplementary material for: Phylogeny‐guided characterization of glycosyltransferases for epothilone glycosylation
Source: Microb Biotechnol. 2019 May 8;12(4):763–74. doi: 10.1111/1751-7915.13421 (PMC6559208; doi:10.1111/1751-7915.13421)
Supplement: Supplementary file 1 — Figure S1. SDS‐PAGE detection of the expression of different GTs. Each of the heterologously expressed GTs contains a His tag. All of the GTs were soluble. M is the protein molecular weight marker, Lane 1, the control of pET 28a vector; Lanes 2–11, the E. coli lysate before induction; Lanes 12–21, the E. coli lysate after induction; Lanes 22–31, the soluble supernatants after induction. Figure S2. SDS‐PAGE detection of the purification of YjiC and YojK proteins. (A). The purification with different concentrations of imidazole. Lanes 1–3, the purification of YjiC with 50 mM imidazole; Lanes 4–7, the purification of YjiC with 100 mM imidazole. Lanes 8–10, the purification of YojK with 50 mM imidazole; Lanes 11–14, the purification of YojK with 100 mM imidazole. (B). The purified YjiC, BsGT‐1, YojK and BsGT‐1. M is the protein molecular weight marker. Figure S3. HPLC detection of the glycosylation products from epothilone A by purified GTs. The control is the standard of epothilone A. The reactions were performed for 2 h of incubation time. Figure S4. 1H NMR (600 MHz) of epothilone A 7‐O‐β‐d glucoside in CD3OD. Figure S5. 13C NMR (150 MHz) of epothilone A 7‐O‐β‐d glucoside in CD3OD. Figure S6. COSY of epothilone A 7‐O‐β‐d glucoside in CD3OD. Figure S7. HSQC of epothilone A 7‐O‐β‐d glucoside in CD3OD. Figure S8. HMBC of epothilone A 7‐O‐β‐d glucoside in CD3OD. Figure S9. (A) Kinetic parameters and curves analysis of high‐active GTs‐catalyzed reactions. Determination of kinetic parameters for epothilone A with saturated UDP‐d‐glucose (10 mM): epothilone A was set as different concentrations from 10–400 μM. Enzyme assays were performed in 50 mM Tris–HCl buffer (pH 7.5) containing 20 μg ml−1 GTs and 10 mM MgCl2 at 37°C for 10 min in triplicate. (B) Kinetic parameters and curves analysis of low‐active GTs‐catalyzed reactions. Determination of kinetic parameters for epothilone A with saturated UDP‐d‐glucose (10 mM): epothilone A was set as different concentrations from 100–4 [file MBT2-12-763-s001.docx]

**Phylogeny-guided Characterization of Glycosyltransferases for Epothilone glycosylation**

Peng Zhang, Zheng Zhang, Zhi-feng Li, Qi Chen, Yao-yao Li, Ya Gong, Xin-jing Yue, Duo-hong Sheng, You-ming Zhang, Changsheng Wu*, Yue-zhong Li*

*State Key Laboratory of Microbial Technology, Institute of Microbial Technology, Shandong University, Qingdao 266237, P.R. China*

Chang-sheng Wu, [wcs0313@gmail.com](mailto:wcs0313@gmail.com).

Yue-zhong Li, [lilab@sdu.edu.cn](mailto:lilab@sdu.edu.cn); Tel. (+86) 532 58631539; ORCID ID, 0000-0001-8336-6638.

***** The corresponding authors.

Running Title: Epothilone glycosyltransferases

**List of figures and tables**

Figure S1, S2, S3, S4, S5, S6, S7, S8, S9, S10, S11

Table S1, S2, S3

**Supplementary Figures**


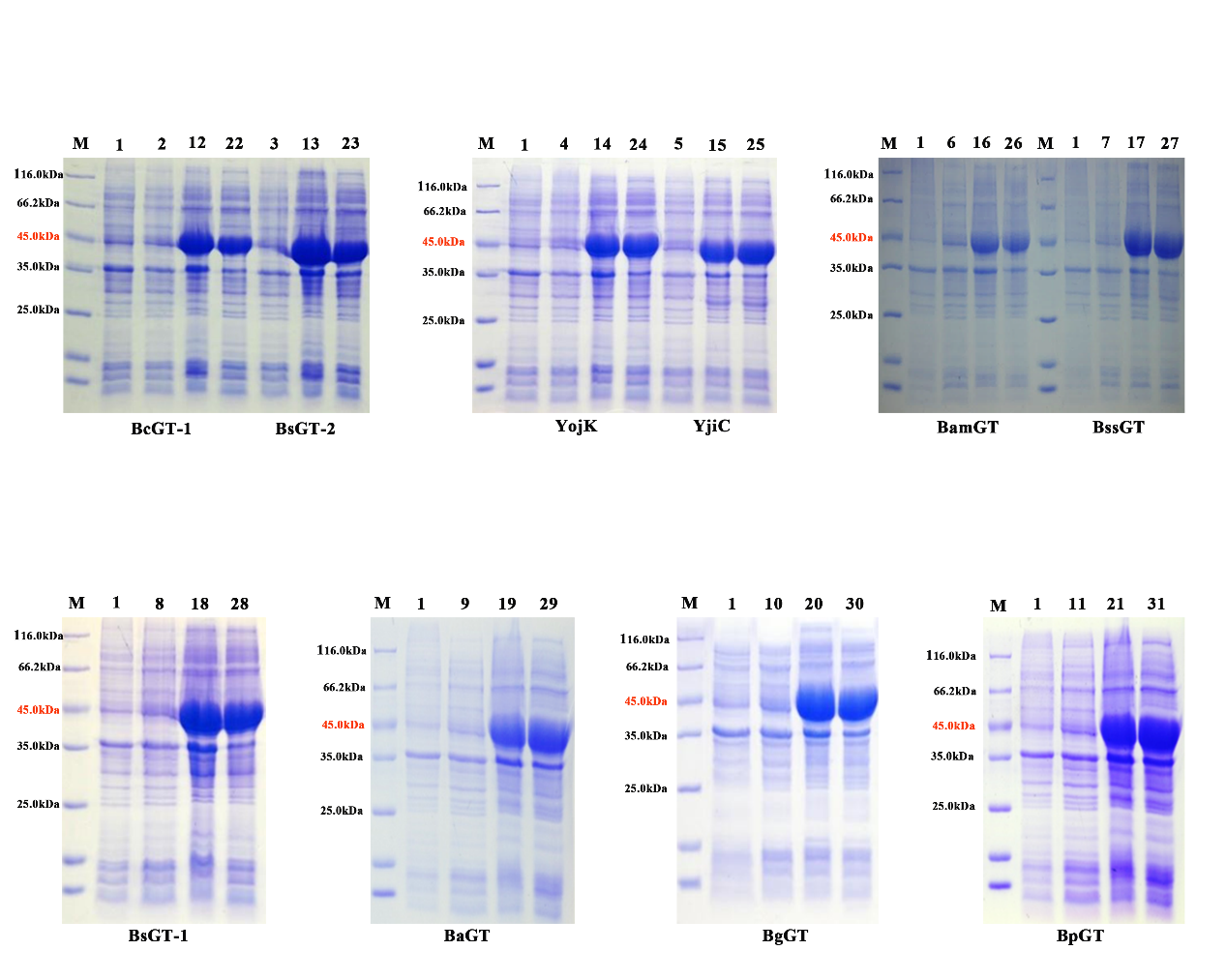


**Figure S1**. SDS-PAGE detection of the expression of different GTs. Each of the heterologously expressed GTs contains a His tag. All of the GTs were soluble. M is the protein molecular weight marker, Lane 1, the control of pET 28a vector; Lanes 2-11, the *E. coli* lysate before induction; Lanes 12-21, the *E. coli* lysate after induction; Lanes 22-31, the soluble supernatants after induction.


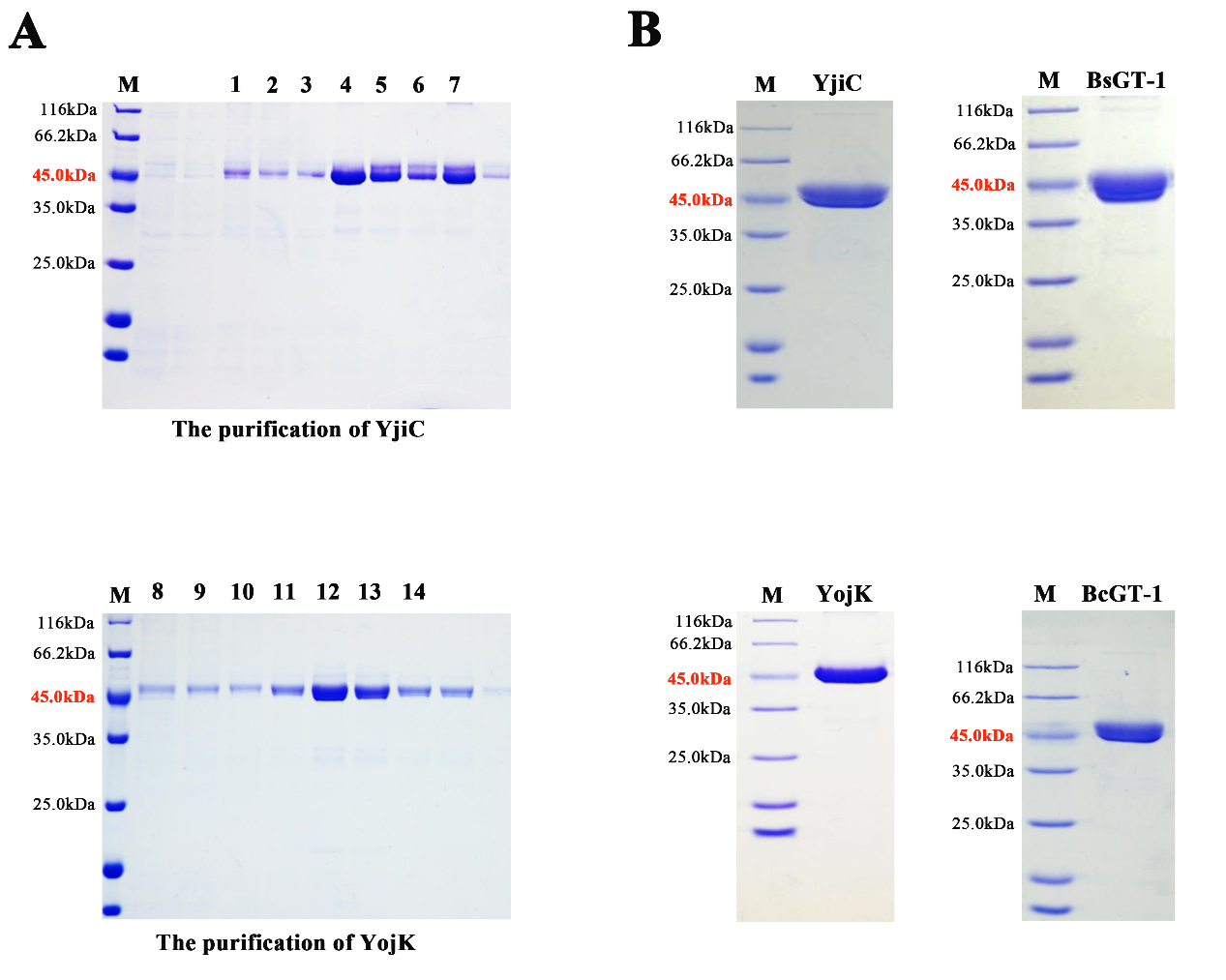


**Figure S2**. SDS-PAGE detection of the purification of YjiC and YojK proteins. (A). The purification with different concentrations of imidazole. Lanes 1-3, the purification of YjiC with 50 mM imidazole; Lanes 4-7, the purification of YjiC with 100 mM imidazole. Lanes 8-10, the purification of YojK with 50 mM imidazole; Lanes 11-14, the purification of YojK with 100 mM imidazole. (B). The purified YjiC, BsGT-1, YojK and BsGT-1. M is the protein molecular weight marker.


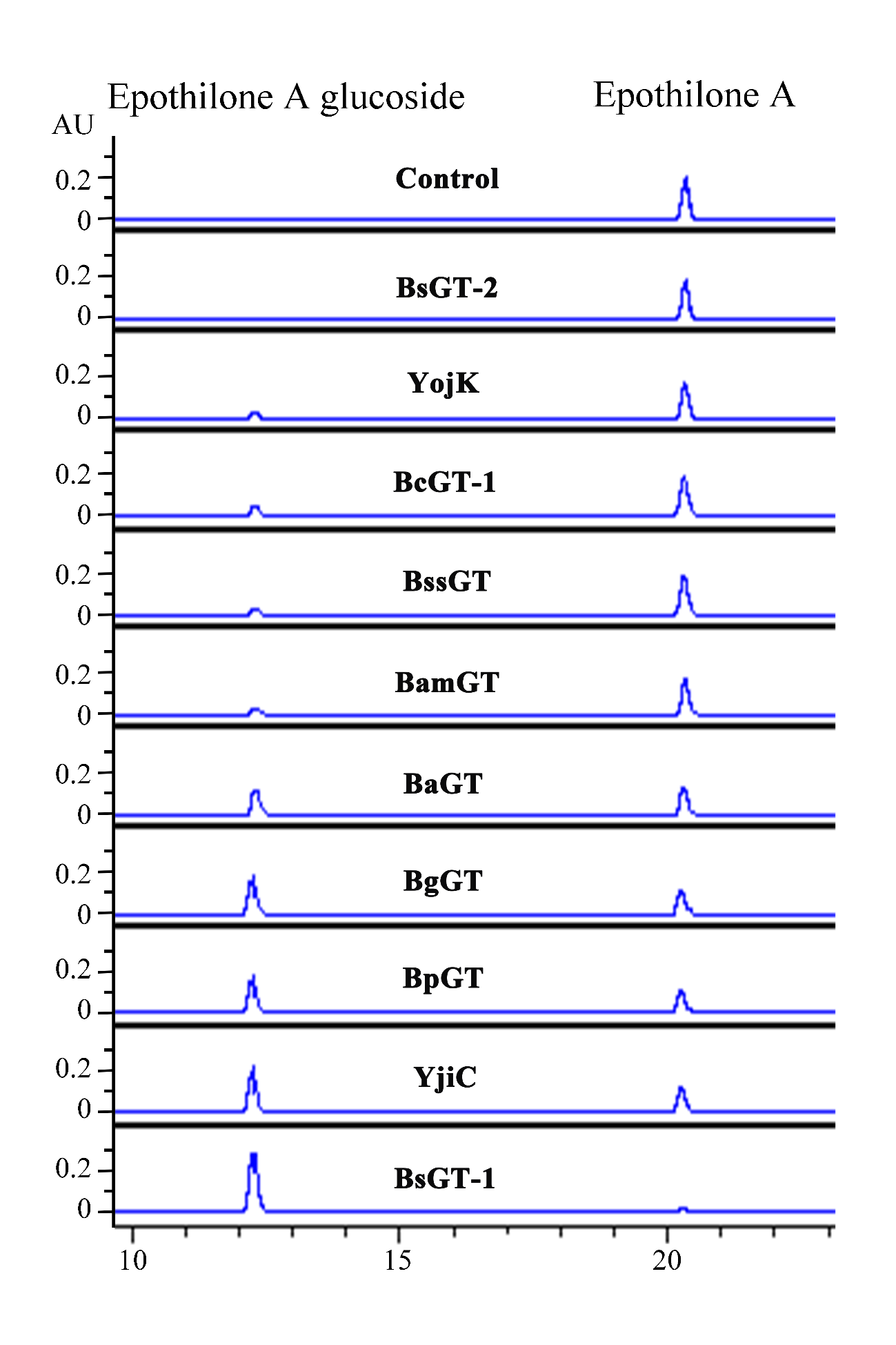


**Figure S3**. HPLC detection of the glycosylation products from epothilone A by purified GTs. The control is the standard of epothilone A. The reactions were performed for 2 h of incubation time.

**Figure S4.** ^1^H NMR (600 MHz) of epothilone A 7-O-β-D glucoside in CD_3_OD


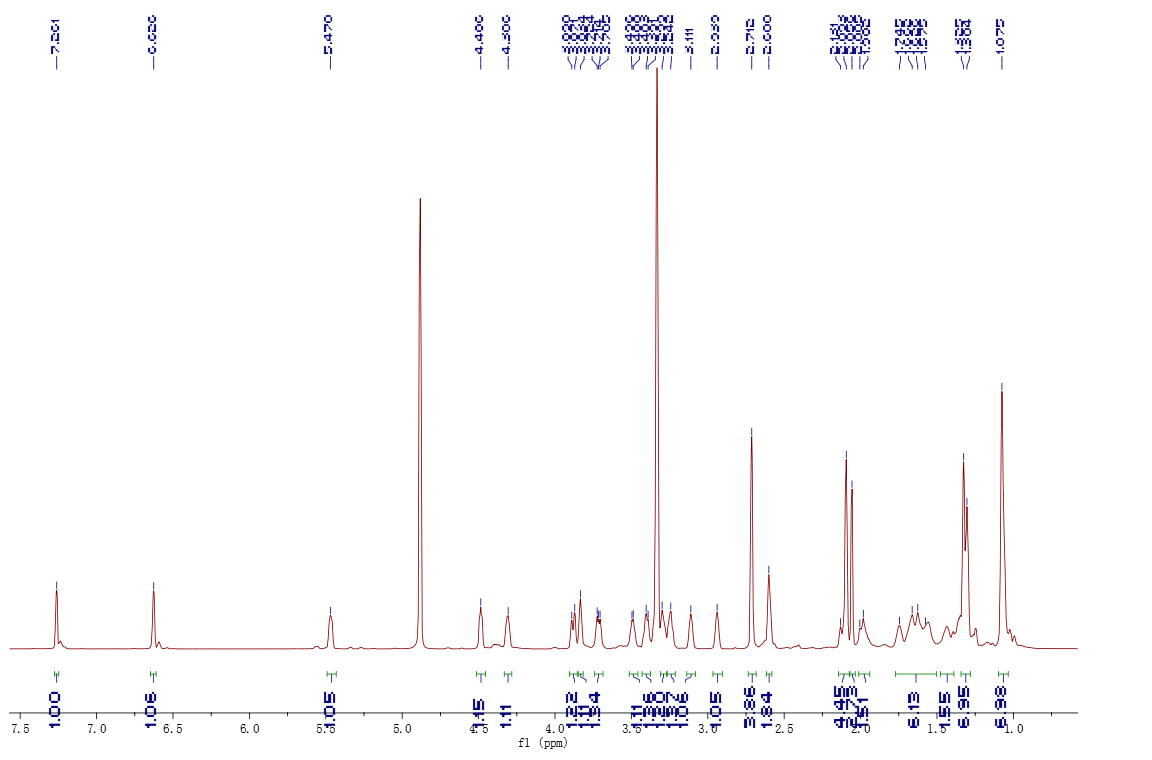


**Figure S5.** ^13^C NMR (150 MHz) of epothilone A 7-O-β-D glucoside in CD_3_OD


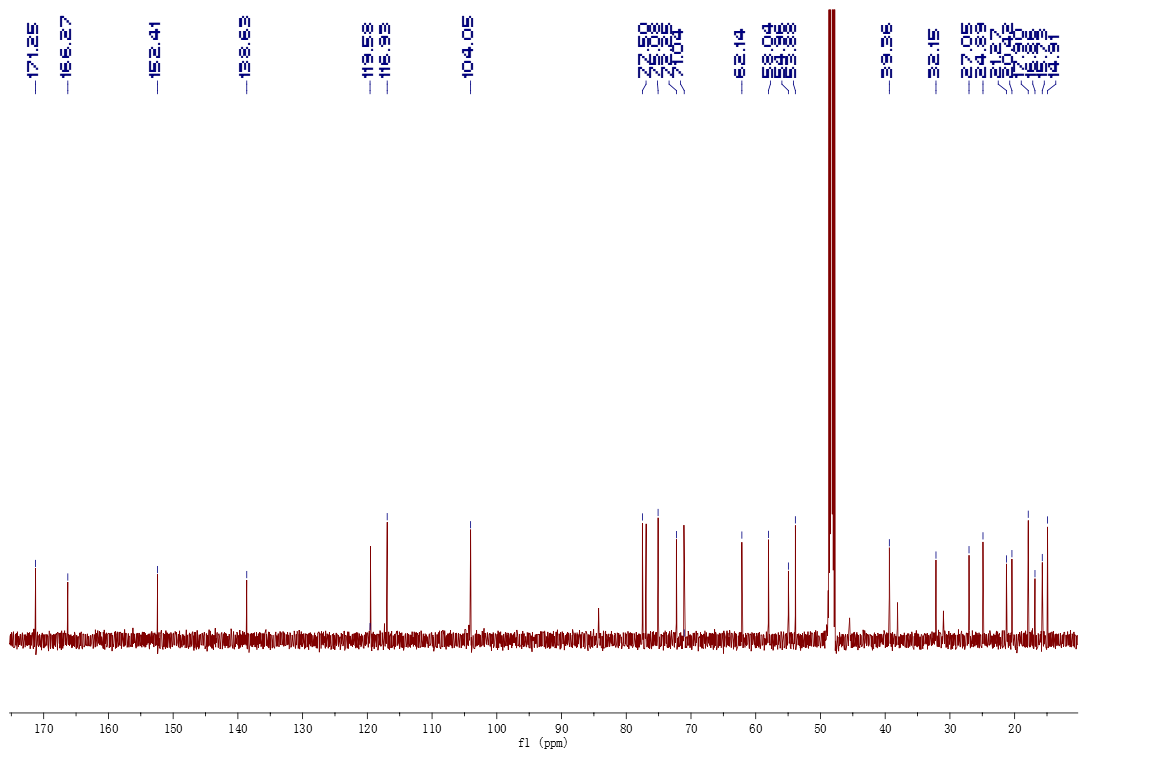


**Figure S6.** COSY of epothilone A 7-O-β-D glucoside in CD_3_OD


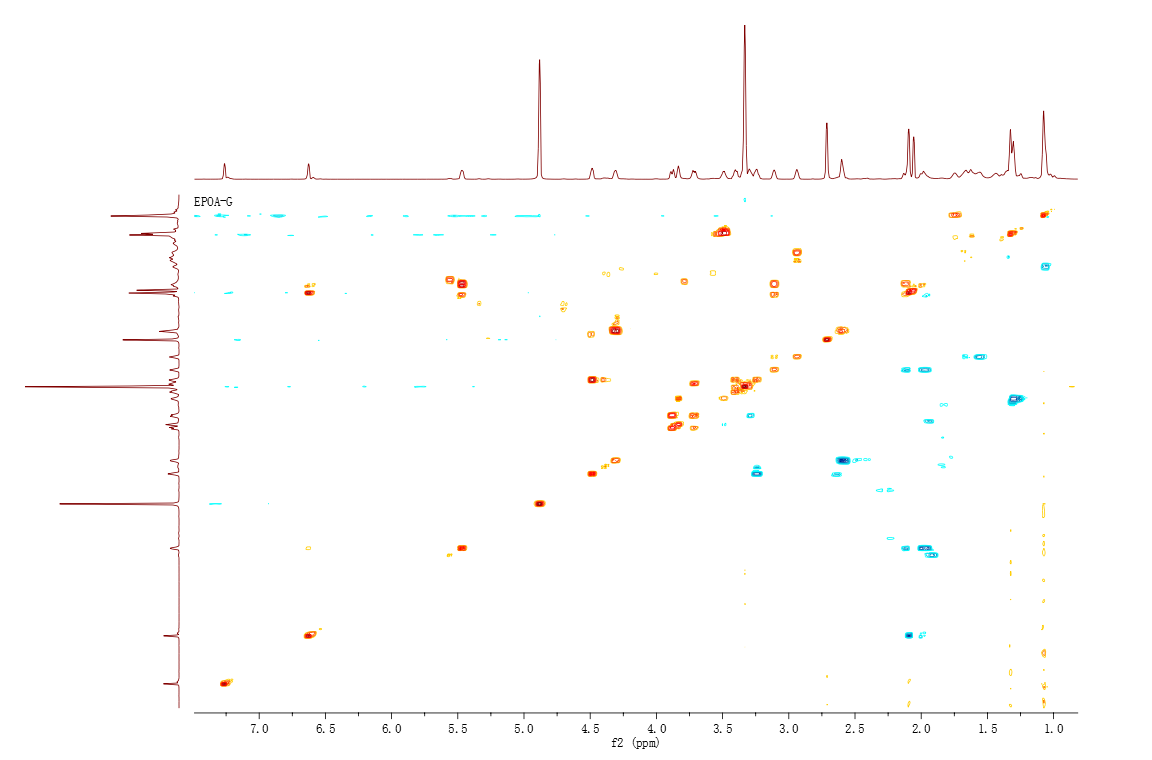


**Figure S7.** HSQC of epothilone A 7-O-β-D glucoside in CD_3_OD


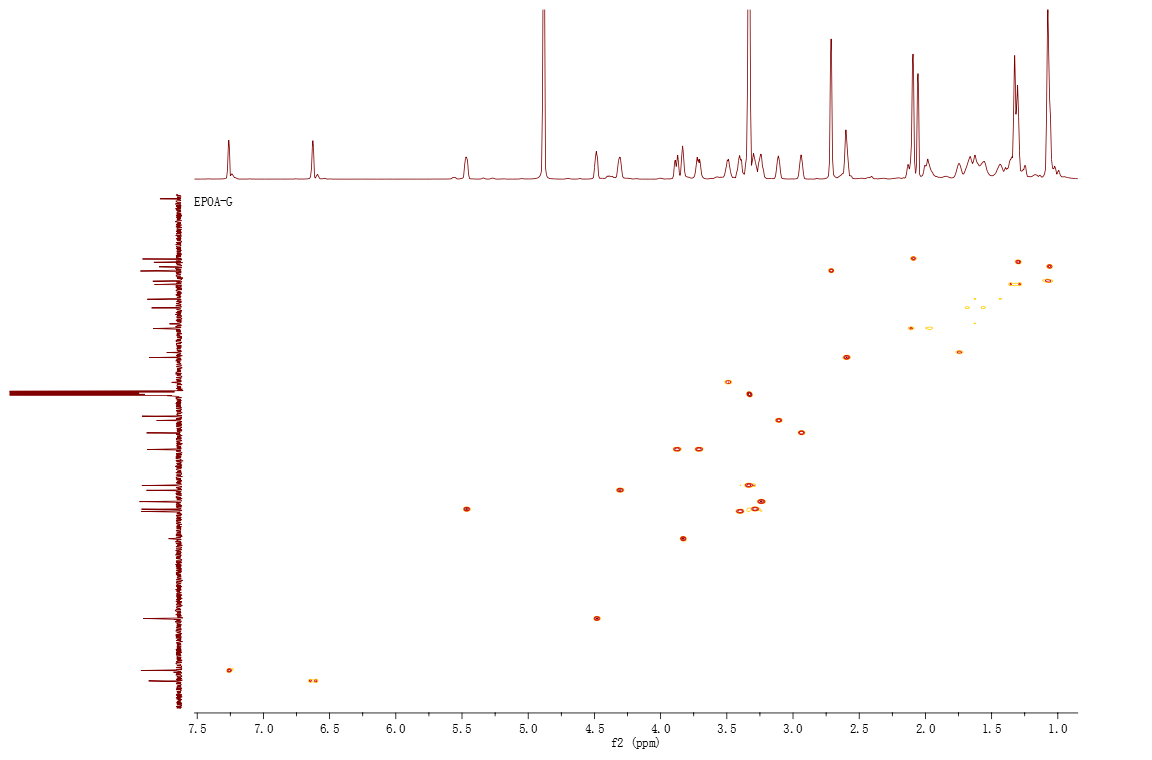


**Figure S8.** HMBC of epothilone A 7-O-β-D glucoside in CD_3_OD


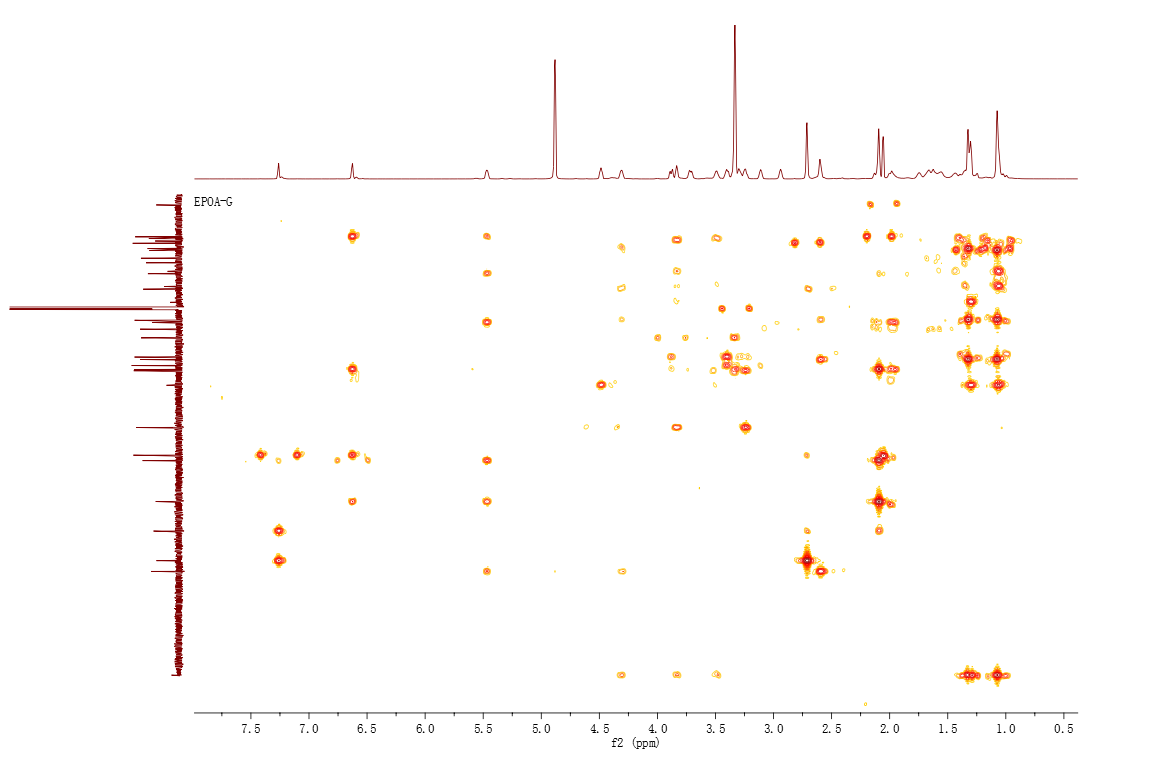


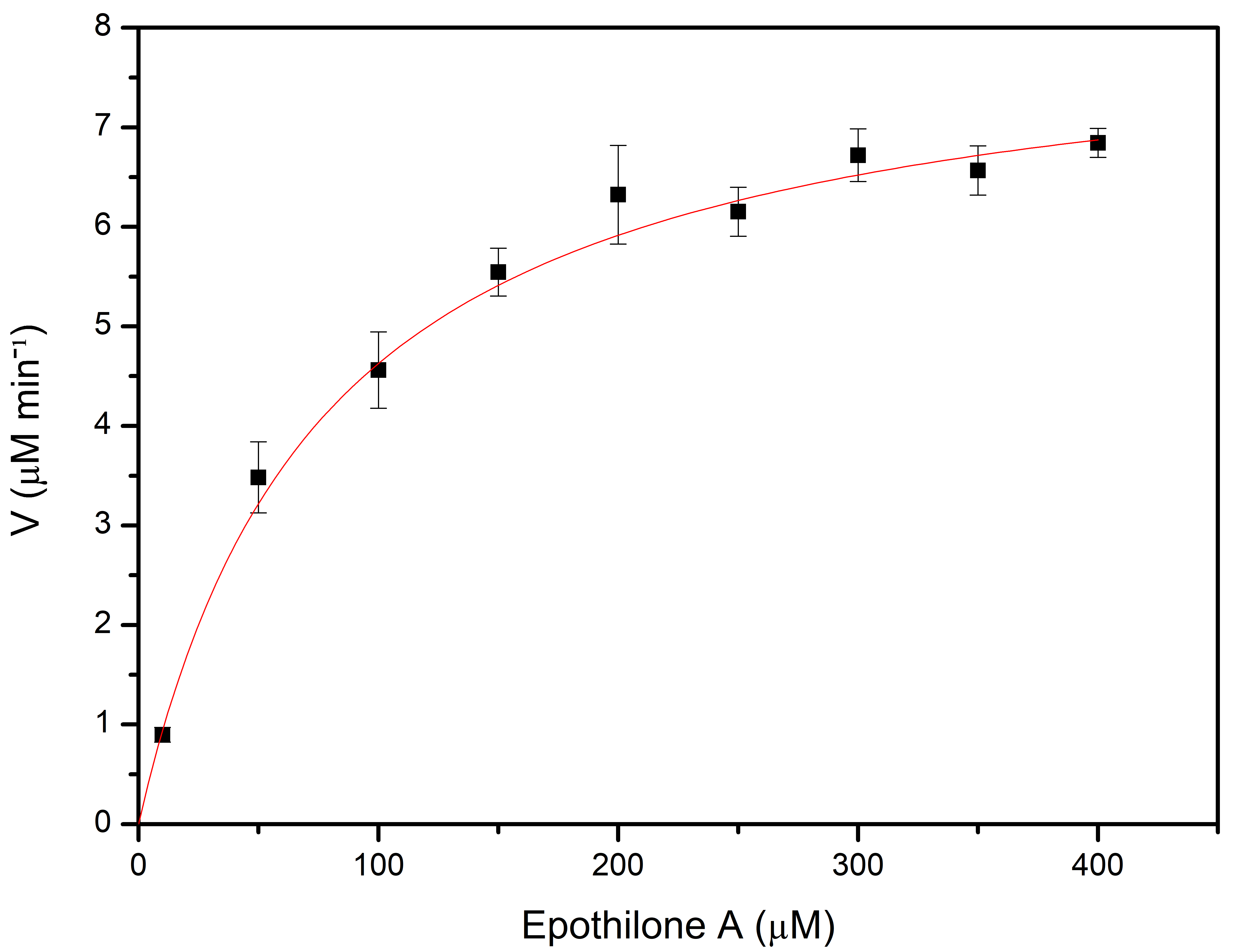

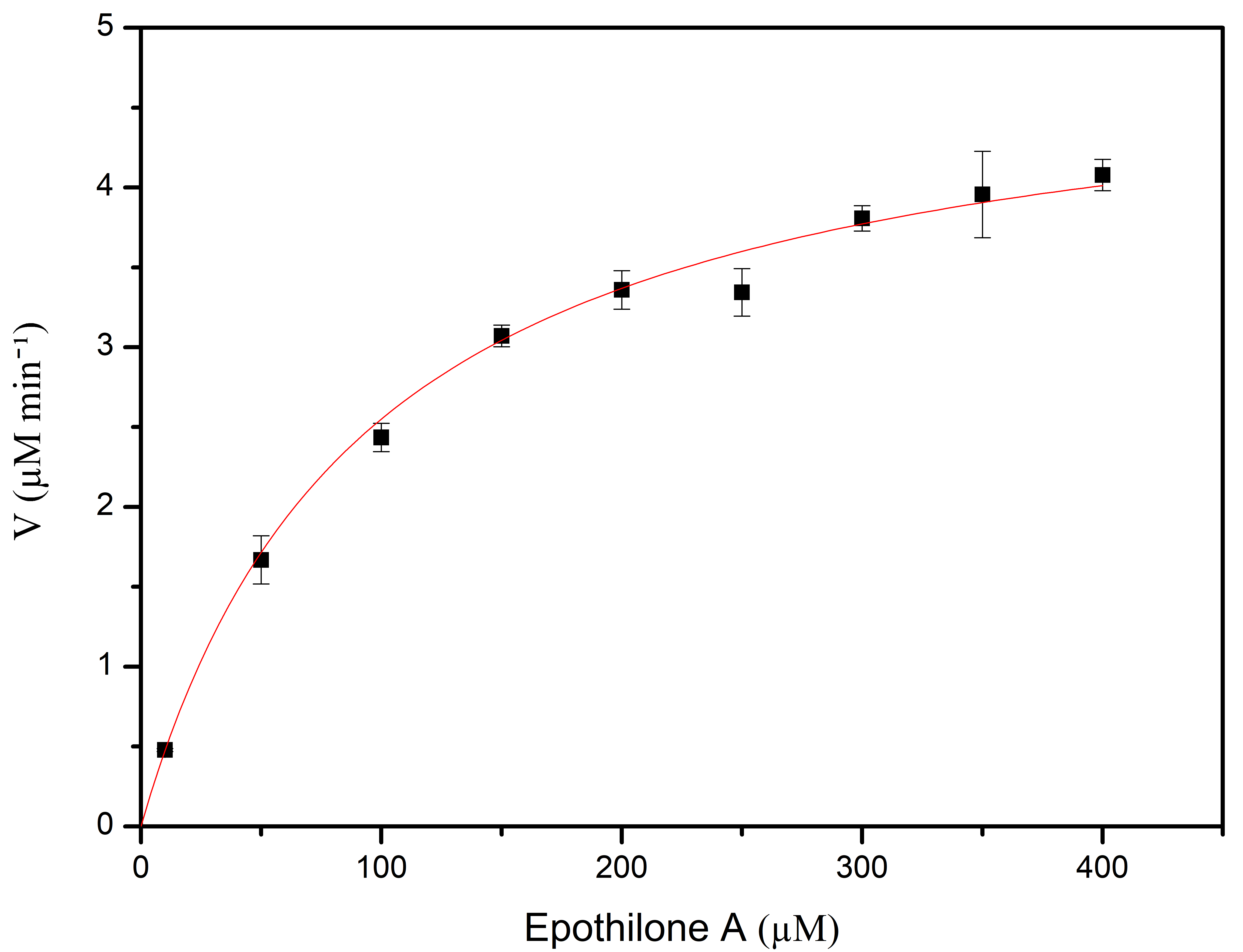


**A.** Michaelis-Menten curve of BsGT-1

**B.** Michaelis-Menten curve of YjiC

*K_M_*= 57.79±4.98 μm

V_max_= 7.77±0.15 μm min^-1^

*k_cat_*= 17.66±0.34 min^-1^

*k_cat_*/*K_M_*= 306.06 min^-1^mm^-1^

*K_M_*= 94.58±2.96 μm

V_max_= 4.96±0.08 μm min^-1^

*k_cat_*= 11.53±0.19 min^-1^

*k_cat_*/*K_M_*= 121.91 min^-1^mm^-1^


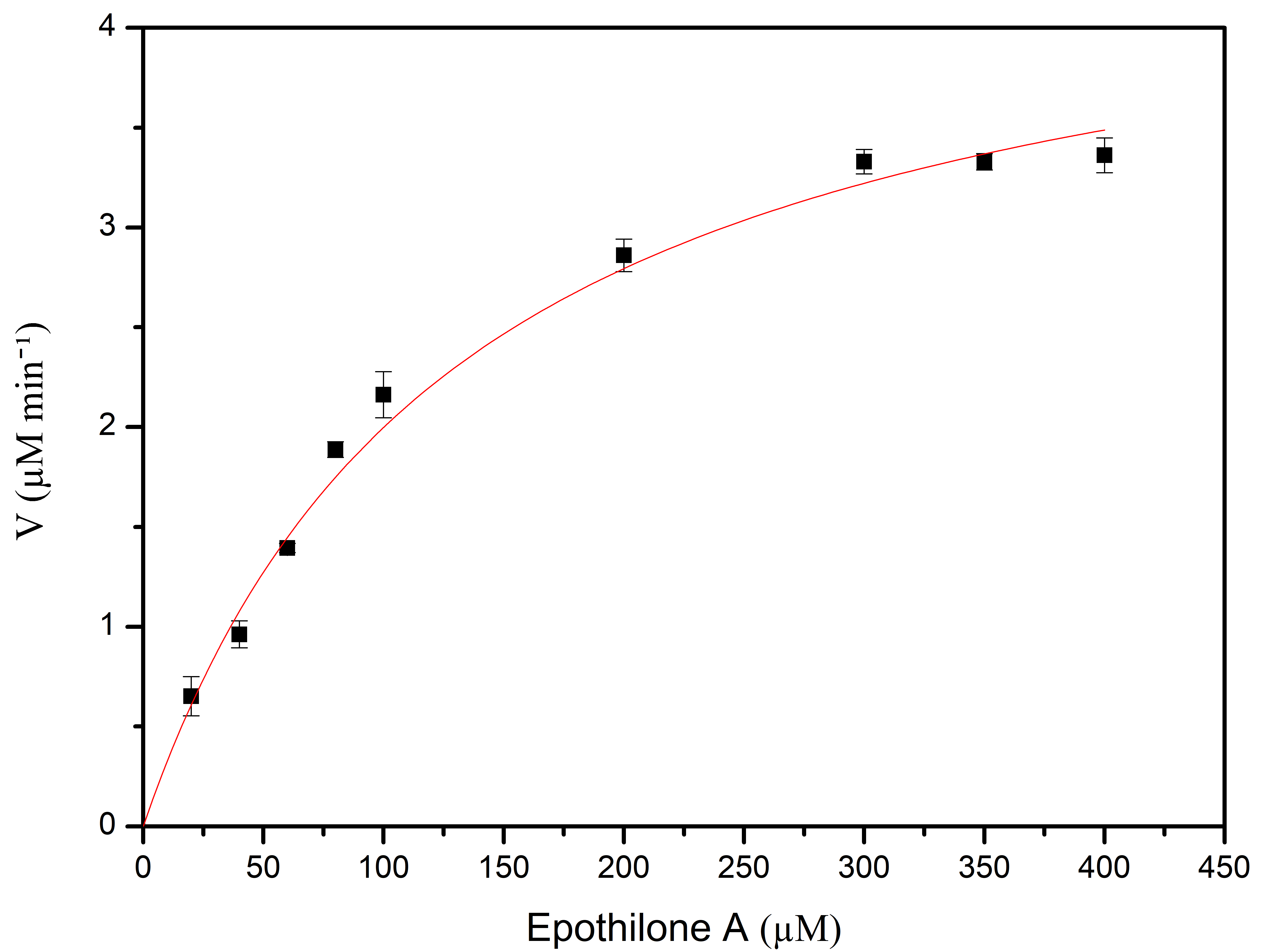

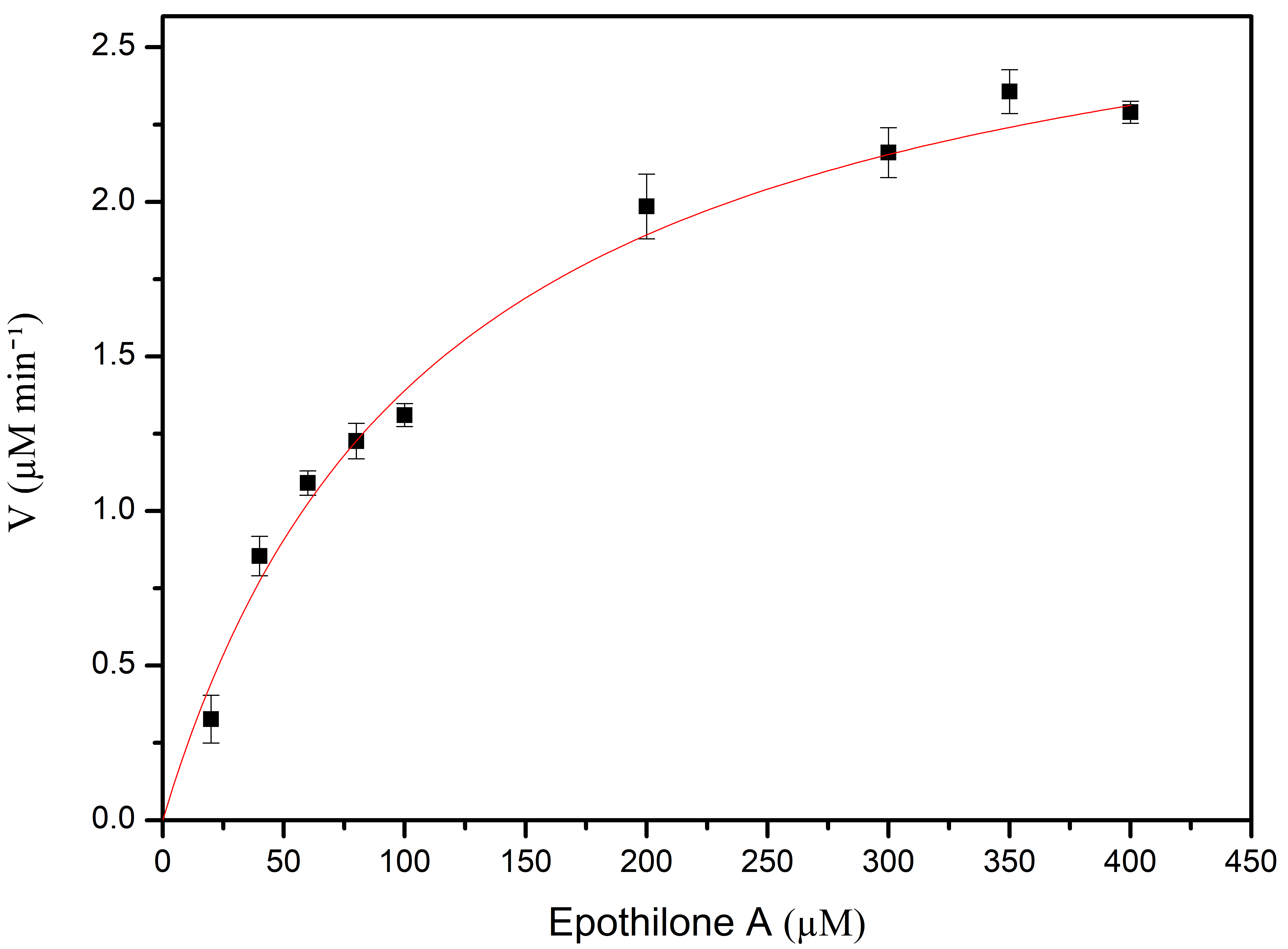


**D.** Michaelis-Menten curve of BgGT

**C.** Michaelis-Menten curve of BaGT


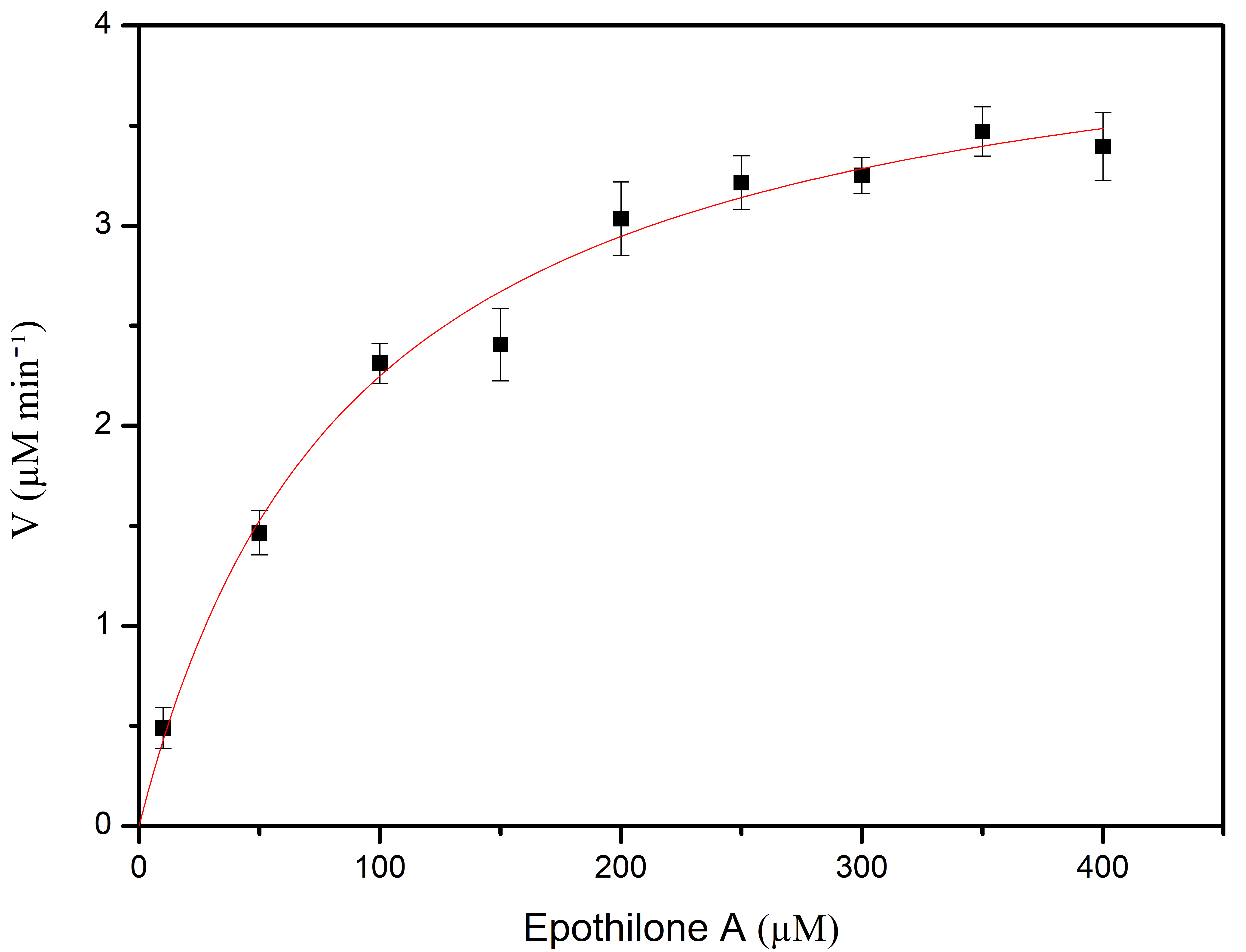


*K_M_*= 89.79±9.02 μm

V_max_= 4.27±0.13 μm min^-1^

*k_cat_*= 9.70±0.30 min^-1^

*k_cat_*/*K_M_*= 108.03 min^-1^mm^-1^

*K_M_*= 132.37±10.91 μm

V_max_= 4.64±0.18 μm min^-1^

*k_cat_*= 10.31±0.40 min^-1^

*k_cat_*/*K_M_*= 77.89 min^-1^mm^-1^

*K_M_*= 113.66±10.55 μm

V_max_= 2.97±0.11 μm min^-1^

*k_cat_*= 6.75±0.25 min^-1^

*k_cat_*/*K_M_*= 59.39 min^-1^mm^-1^

**E.** Michaelis-Menten curve of BpGT

**Figure S9**. **(A)** Kinetic parameters and curves analysis of high-active GTs-catalyzed reactions. Determination of kinetic parameters for epothilone A with saturated UDP-D-glucose (10 mM): epothilone A was set as different concentrations from 10-400 μM. Enzyme assays were performed in 50 mM Tris-HCl buffer (pH 7.5) containing 20 μg/ml GTs and 10 mM MgCl_2_ at 37°C for 10 min in triplicate.


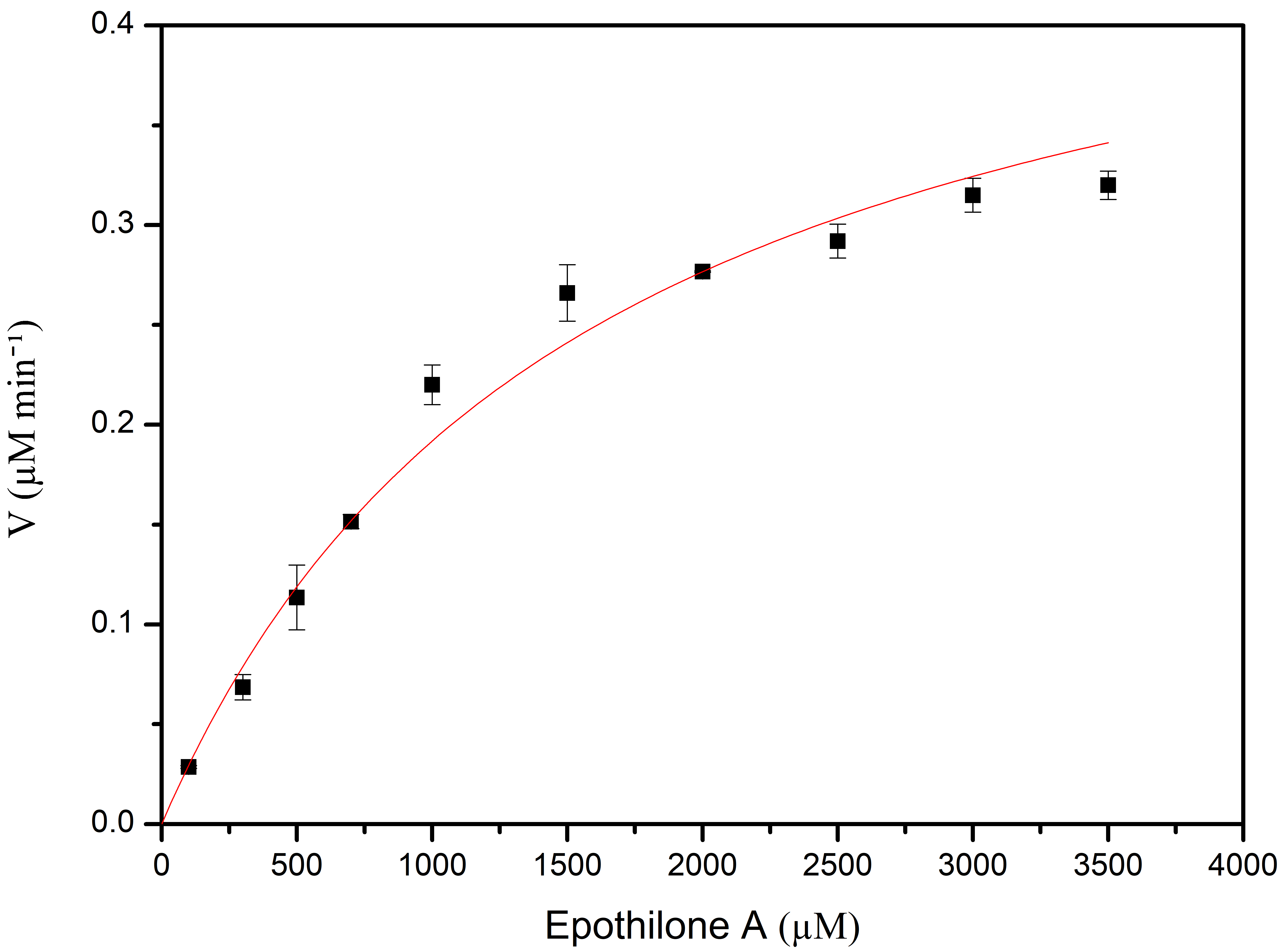

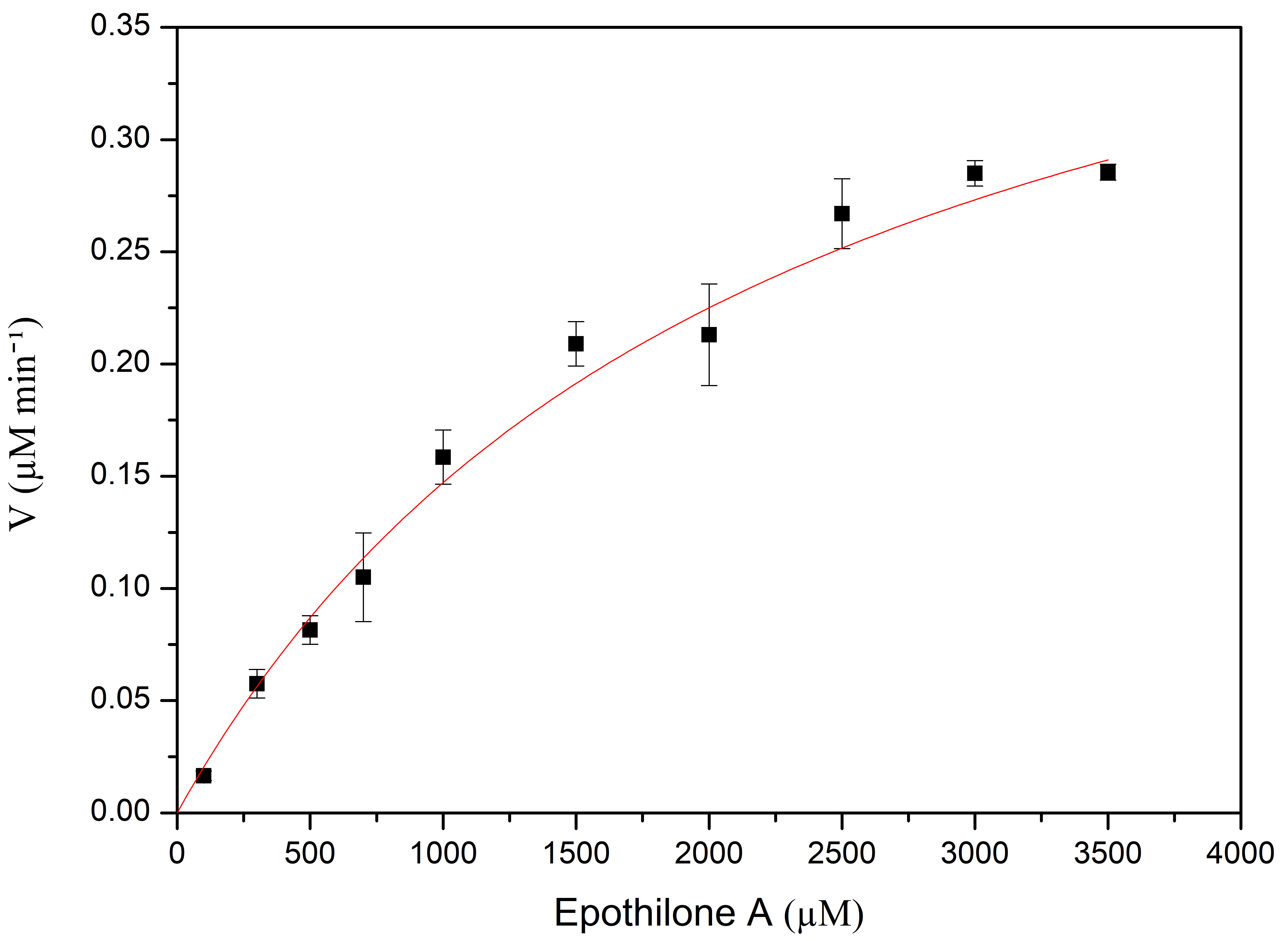

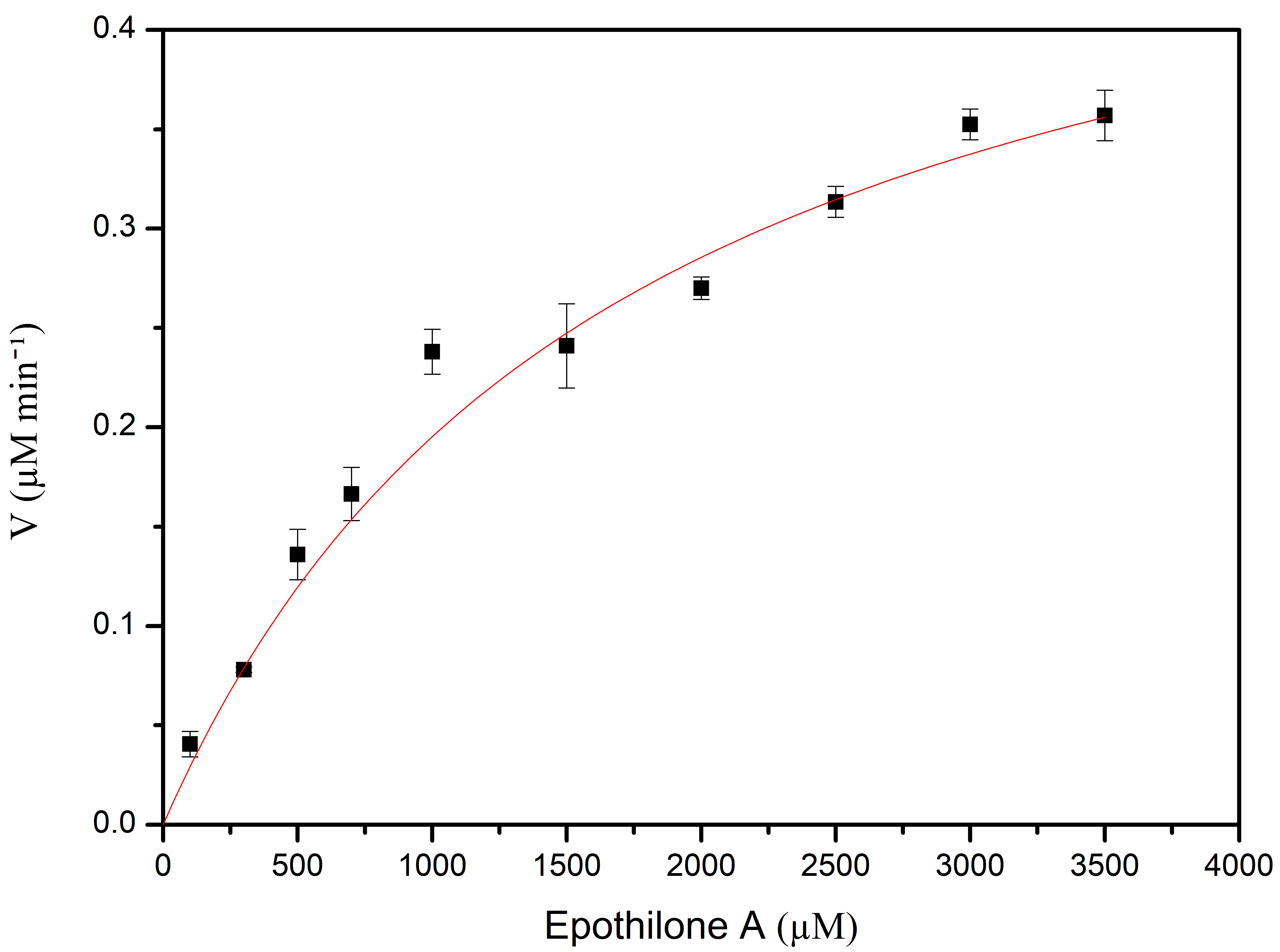

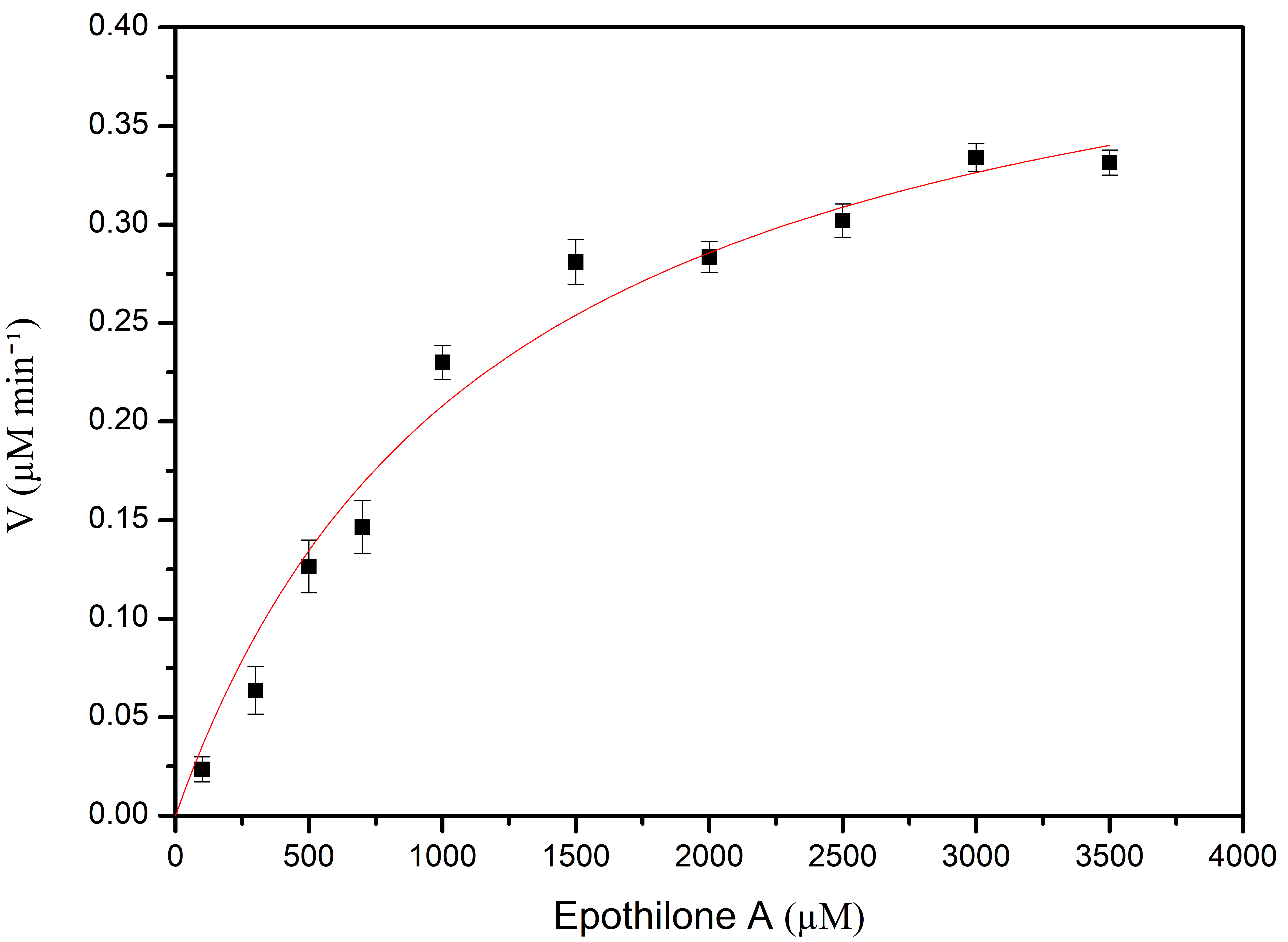


*K_M_*= 1196.12±214.98 μm

V_max_= 0.46±0.03 μm min^-1^

*k_cat_*= 1.07±0.07 min^-1^

*k_cat_*/*K_M_*= 0.89 min^-1^mm^-1^

**F.** Michaelis-Menten curve of BssGT

*K_M_*= 1585.26±116.74 μm

V_max_= 0.50±0.02 μm min^-1^

*k_cat_*= 1.14±0.05 min^-1^

*k_cat_*/*K_M_*= 0.72 min^-1^mm^-1^

**G.** Michaelis-Menten curve of BamGT

*K_M_*= 2245.74±311.96 μm

V_max_= 0.48±0.03 μm min^-1^

*k_cat_*= 1.09±0.07 min^-1^

*k_cat_*/*K_M_*= 0.50 min^-1^mm^-1^

*K_M_*= 1720.77±161.35 μm

V_max_= 0.53±0.03 μm min^-1^

*k_cat_*= 1.23±0.07 min^-1^

*k_cat_*/*K_M_*= 0.71 min^-1^mm^-1^

**H.** Michaelis-Menten curve of YojK

**I.** Michaelis-Menten curve of BcGT-1

**Figure S9**. **(B)** Kinetic parameters and curves analysis of low-active GTs-catalyzed reactions. Determination of kinetic parameters for epothilone A with saturated UDP-D-glucose (10 mM): epothilone A was set as different concentrations from 100-4000 μM. Enzyme assays were performed in 50 mM Tris-HCl buffer (pH 7.5) containing 20 μg/ml GTs and 10 mM MgCl_2_ at 37°C for 10 min in triplicate.


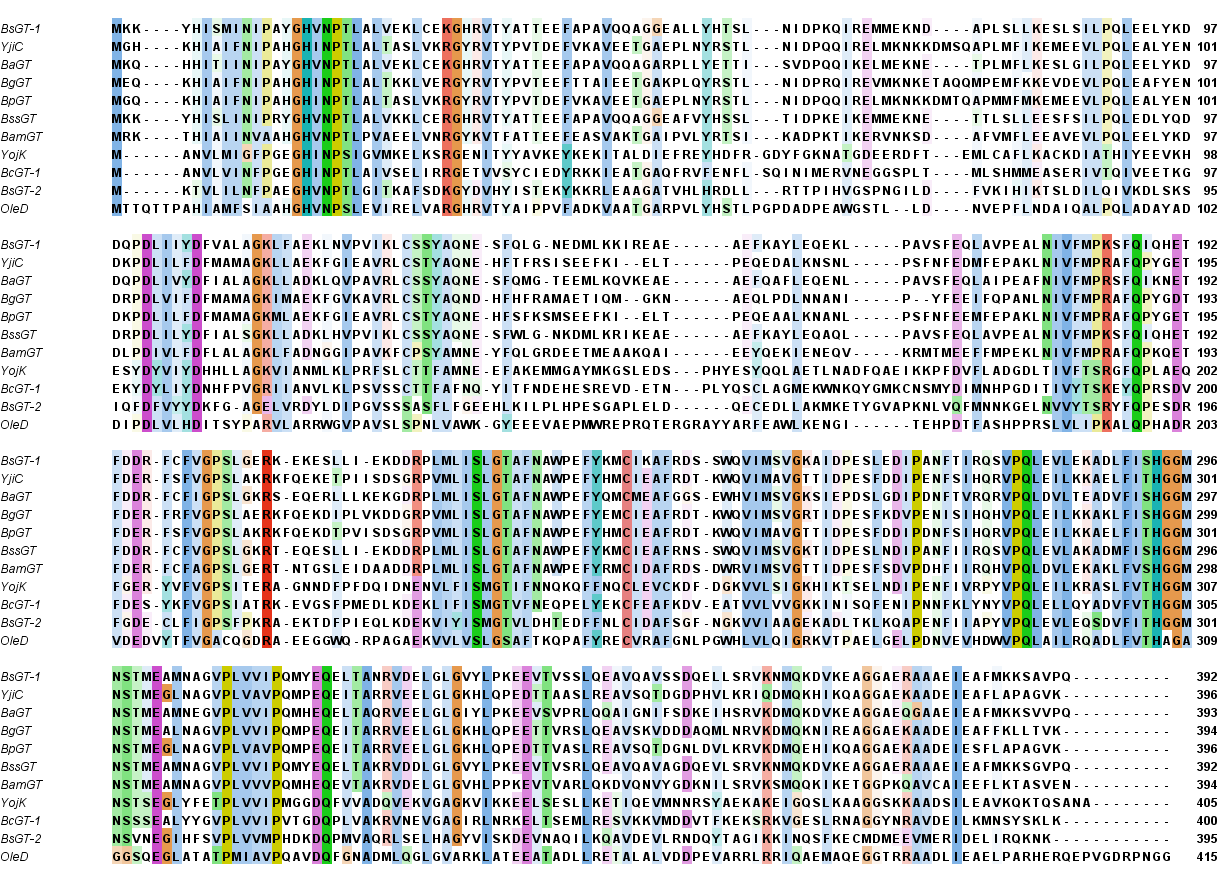


**Figure S10**. Multiple sequence alignment of the ten assayed GTs with OleD as reference. The sites of L54, Q66, P77 and K82 (based on BsGT-1) are highlighted yellow in five high active GTs and two low active GTs.


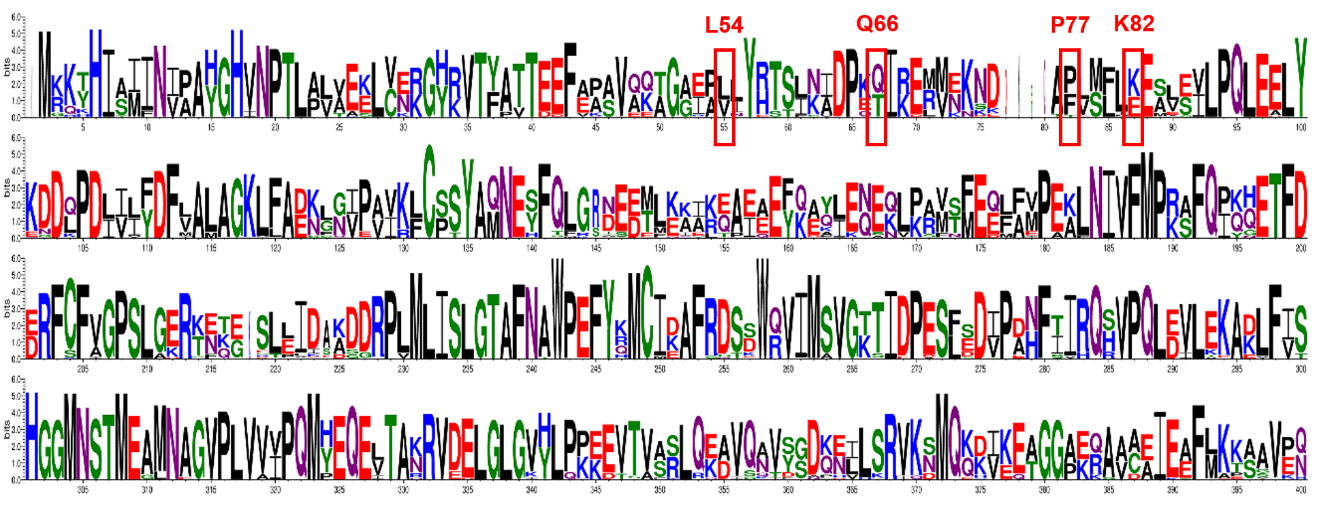


**Figure S11**. The WebLogo shows the amino acid residues comparison of the 161 GT members in the YjiC-subbranch. The four amino acid positions (L54, Q66, P77 and K82, based on BsGT-1) were marked with red boxes and their actual locations (based on BsGT-1) were numbered.

**Supplementary Tables**

**Table S1.** 161 numbers in the YjiC-subbranch GTs.

| **No.** | **Organism** | **Protein Name** | **GenBank** |
| --- | --- | --- | --- |
| 1 | *Bacillus amyloliquefaciens B15* | A1R12_06055 | AMR52458.1 |
| 2 | *Bacillus amyloliquefaciens CC178* | U471_12310 | AGZ55937.1 |
| 3 | *Bacillus amyloliquefaciens DSM 7* | BAMF_1309 (YjiC) | CBI42435.1 |
| 4 | *Bacillus amyloliquefaciens IT-45* | KSO_013425 | AGF28174.1 |
| 5 | *Bacillus amyloliquefaciens KHG19* | KHU1_1033 | AJK64999.1 |
| 6 | *Bacillus amyloliquefaciens L-H15* | SB45_05825 | AJH23526.1 |
| 7 | *Bacillus amyloliquefaciens L-S60* | XM40_05850 | AKD21753.1 |
| 8 | *Bacillus amyloliquefaciens LFB112* | U722_06380 | AHC41738.1 |
| 9 | *Bacillus amyloliquefaciens LL3* | LL3_01317 (YjiC) | AEB62858.1 |
| 10 | *Bacillus amyloliquefaciens LM2303* | BSF20_00840 | APH47054.1 |
| 11 | *Bacillus amyloliquefaciens MBE1283* | AVM03_00645 | ALV00941.1 |
| 12 | *Bacillus amyloliquefaciens MT45* | WV34_06630 | ASF28447.1 |
| 13 | *Bacillus amyloliquefaciens RD7-7* | BARD7_01182 | AOC90652.1 |
| 14 | *Bacillus amyloliquefaciens S499* | AS588_09210 | AMP32199.1 |
| 15 | *Bacillus amyloliquefaciens SRCM101267* | S101267_01421 | ARW38509.1 |
| 16 | *Bacillus amyloliquefaciens subsp. plantarum AH159-1* | ORF | BAP05458.1 |
| 17 | *Bacillus amyloliquefaciens TA208* | BAMTA208_11035 (YjiC) | AEB24372.1 |
| 18 | *Bacillus amyloliquefaciens UMAF6614* | BAMY6614_10110 | AMQ73662.1 |
| 19 | *Bacillus amyloliquefaciens UMAF6639* | BAMY6639_17675 | AMQ70722.1 |
| 20 | *Bacillus amyloliquefaciens WS-8* | BSO20_05785 | APQ49603.1 |
| 21 | *Bacillus amyloliquefaciens XH7* | BAXH7_02259 (YjiC) | AEK89389.1 |
| 22 | *Bacillus amyloliquefaciens Y14* | BAMY_06000 | APB81683.1 |
| 23 | *Bacillus amyloliquefaciens Y2* | MUS_1310 (YjiC) | AFJ61330.1 |
| 24 | *Bacillus atrophaeus 1942* | BATR1942_03760 | ADP31706.1 |
| 25 | *Bacillus atrophaeus GQJK17* | BaGK_06920 | ASS70701.1 |
| 26 | *Bacillus atrophaeus NRS 1221A* | TD68_03320 | AJF84512.1 |
| 27 | *Bacillus atrophaeus SRCM101359* | S101359_01288 | ARW06295.1 |
| 28 | *Bacillus atrophaeus subsp. globigii BSS* | DJ95_680 (OleI) | AIK47254.1 |
| 29 | *Bacillus atrophaeus UCMB-5137* | D068_cds12520 (YjiC) | AKL83970.1 |
| 30 | *Bacillus gibsonii FJAT-10019* | BGM20_00385 | AOL29161.1 |
| 31 | *Bacillus glycinifermentans* | BGLY_2157 | SCA85980.1 |
| 32 | *Bacillus licheniformis BL1202* | BL1202_02098 | AOP15046.1 |
| 33 | *Bacillus licheniformis DSM 13 = ATCC 14580* | UDP-Glc: isoflavonoid β-glucosyltransferase (YjiC;BLi01948;BL00446) | AAU40842.1 |
| 34 | *Bacillus licheniformis HRBL-15TDI7* | AB684_09185 | AMR10347.1 |
| 35 | *Bacillus licheniformis SCCB 37* | B37_01466 (Oled_2) | ARC73518.1 |
| 36 | *Bacillus licheniformis SCDB 14* | B14_02514 (Oled_1) | ARC65512.1 |
| 37 | *Bacillus licheniformis SCDB 34* | B34_00286 (Oled_1) | ARC67729.1 |
| 38 | *Bacillus licheniformis SCK B11* | BaDB11_04455 (Oled_3) | ARC63018.1 |
| 39 | *Bacillus licheniformis SRCM100141* | S100141_01333 | ARW42655.1 |
| 40 | *Bacillus licheniformis SRCM101441* | S101441_01452 | ARW31001.1 |
| 41 | *Bacillus licheniformis SVD1* | ORF | BAL45952.1 |
| 42 | *Bacillus licheniformis WX-02* | MUY_001965 (YjiC) | AKQ73097.1 |
| 43 | *Bacillus licheniformis ZSP1* | glycosyltransferase (Gtzs1) | AKA94108.1 |
| 44 | *Bacillus paralicheniformis ATCC 9945a* | BaLi_c19830 (YjiC) | AGN36351.1 |
| 45 | *Bacillus paralicheniformis BL-09* | SC10_B2orf02948 | AJO18201.1 |
| 46 | *Bacillus paralicheniformis MDJK30* | BLMD_09670 | ARA85718.1 |
| 47 | *Bacillus sonorensis SRCM101395* | S101395_02951 | ASB89458.1 |
| 48 | *Bacillus sp. 1s-1* | CJO35_09630 | ASV15407.1 |
| 49 | *Bacillus sp. 275* | BZ167_03355 | AQP95122.1 |
| 50 | *Bacillus sp. BH072* | OY17_09250 | AJE78263.1 |
| 51 | *Bacillus sp. BS34A* | BS34A_13530 (YjiC) | CEJ76789.1 |
| 52 | *Bacillus sp. FJAT-14266* | BGM23_15465 | AOL27923.1 |
| 53 | *Bacillus sp. H15-1* | BSZ43_09545 | APJ27020.1 |
| 54 | *Bacillus sp. JS* | MY9_1334 | AFI27871.1 |
| 55 | *Bacillus sp. LM 4-2* | BsLM_1284 | AKE23083.1 |
| 56 | *Bacillus sp. MD-5* | CDO84_05675 | ASB60493.1 |
| 57 | *Bacillus sp. Pc3* | SB24_03660 | AJC27216.1 |
| 58 | *Bacillus sp. SDLI1* | AUL54_04470 | AME05659.1 |
| 59 | *Bacillus sp. YP1* | QF06_05090 | AJO57847.1 |
| 60 | *Bacillus subtilis 29R7-12* | BKP58_05480 | API95393.1 |
| 61 | *Bacillus subtilis 3.34* | glycosyltransferase (Yjic1) | AGC59683.1 |
| 62 | *Bacillus subtilis ATCC 13952* | KS08_06275 | AIW33263.1 |
| 63 | *Bacillus subtilis ATCC 19217* | KS07_06040 | AIW37049.1 |
| 64 | *Bacillus subtilis B-1* | MA22_09065 | AIU76654.1 |
| 65 | *Bacillus subtilis BEST7003* | BEST7003_1216 (YjiC) | BAM57417.1 |
| 66 | *Bacillus subtilis Bs-115* | B7470_18480 | ARI87885.1 |
| 67 | *Bacillus subtilis Bs-916* | KO64_06130 | AIW32013.1 |
| 68 | *Bacillus subtilis BS16045* | BS16045_01308 | AOL97026.1 |
| 69 | *Bacillus subtilis BS38* | BSBS38_01400 | AOR97680.1 |
| 70 | *Bacillus subtilis BS49* | BS49_13540 (YjiC) | CEI56366.1 |
| 71 | *Bacillus subtilis BSn5* | BSn5_18070 | ADV96222.1 |
| 72 | *Bacillus subtilis CW14* | BCV50_06150 | ARV44626.1 |
| 73 | *Bacillus subtilis DKU_NT_02* | CJZ70_13470 | ASU99266.1 |
| 74 | *Bacillus subtilis DKU_NT_03* | CJZ71_19325 | ASV04094.1 |
| 75 | *Bacillus subtilis GQJK2* | BSK2_06615 | ARB36618.1 |
| 76 | *Bacillus subtilis HJ0-6* | BSHJ0_01273 | AOA53845.1 |
| 77 | *Bacillus subtilis HJ5* | AW03_012230 | AKD34615.1 |
| 78 | *Bacillus subtilis HRBS-10TDI13* | A4A60_07130 | AOS67438.1 |
| 79 | *Bacillus subtilis J-5* | BHE96_06195 | APH35179.1 |
| 80 | *Bacillus subtilis KCTC 1028* | O7A_06815 | AKC46764.1 |
| 81 | *Bacillus subtilis KCTC1022* | UDP-glycosyltransferase | ANP92054.1 |
| 82 | *Bacillus subtilis KH2* | BSR08_03065 | API41552.1 |
| 83 | *Bacillus subtilis MJ01* | BAX60_12285 | APH68106.1 |
| 84 | *Bacillus subtilis NCIB 3610* | B4U62_06770 | AQZ90114.1 |
| 85 | *Bacillus subtilis PS832* | QX56_06815 | AIY96822.1 |
| 86 | *Bacillus subtilis PY79* | U712_06330 | AHA77213.1 |
| 87 | *Bacillus subtilis QB928* | B657_12220 (YjiC) | AFQ57142.1 |
| 88 | *Bacillus subtilis SG6* | OB04_01317 (Oled_2) | AIX06979.1 |
| 89 | *Bacillus subtilis SR1* | CDA59_18050 | ASC84208.1 |
| 90 | *Bacillus subtilis subsp. globigii ATCC 49760* | A1D11_13415 | AMR63355.1 |
| 91 | *Bacillus subtilis subsp. inaquosorum DE111* | AN935_06335 | AMA51908.1 |
| 92 | *Bacillus subtilis subsp. natto BEST195* | BSNT_02071 (YjiC) | BAI84807.2 |
| 93 | *Bacillus subtilis subsp. natto CGMCC 2108* | AWV81_06850 | AMK71852.1 |
| 94 | *Bacillus subtilis subsp. spizizenii NRS 231* | SD85_06485 | AJD35886.1 |
| 95 | *Bacillus subtilis subsp. spizizenii str. W23* | BSUW23_06215 (YjiC) | ADM37292.1 |
| 96 | *Bacillus subtilis subsp. spizizenii TU-B-10* | GYO_1529 | AEP86175.1 |
| 97 | *Bacillus subtilis subsp. subtilis 168G* | BFI33_06805 | AOA10519.1 |
| 98 | *Bacillus subtilis subsp. subtilis 3NA* | RP72_06700 | AJE93890.1 |
| 99 | *Bacillus subtilis subsp. subtilis 6051-HGW* | BSU6051_12220 (YjiC) | AGG60578.1 |
| 100 | *Bacillus subtilis subsp. subtilis BSD-2* | AT706_11995 | ALS82618.1 |
| 101 | *Bacillus subtilis subsp. subtilis CU1050* | AWM80_06685 | AMB23637.1 |
| 102 | *Bacillus subtilis subsp. subtilis delta6* | A8O17_06525 | ANJ30268.1 |
| 103 | *Bacillus subtilis subsp. subtilis KCTC 3135* | AS891_17425 | ANX08934.1 |
| 104 | *Bacillus subtilis subsp. subtilis QB5412* | BHY07_06805 | AOT47486.1 |
| 105 | *Bacillus subtilis subsp. subtilis QB5413* | BH660_06820 | AOT51697.1 |
| 106 | *Bacillus subtilis subsp. subtilis SRCM100333* | S100333_01407 | ASB69300.1 |
| 107 | *Bacillus subtilis subsp. subtilis SRCM100757* | S100757_01380 | ARW02311.1 |
| 108 | *Bacillus subtilis subsp. subtilis SRCM100761* | S100761_01388 | ASB56717.1 |
| 109 | *Bacillus subtilis subsp. subtilis SRCM101392* | S101392_01313 | ASB92786.1 |
| 110 | *Bacillus subtilis subsp. subtilis SRCM101444* | S101444_01386 | ARV98234.1 |
| 111 | *Bacillus subtilis subsp. subtilis str. 168* | BSU12220 (YjiC) | AAC46318.1 |
| 112 | *Bacillus subtilis subsp. subtilis str. AG1839* | BSUB_01330 (YjiC) | AIC43848.1 |
| 113 | *Bacillus subtilis subsp. subtilis str. BAB-1* | I653_06230 | AGI28502.1 |
| 114 | *Bacillus subtilis subsp. subtilis str. BSP1* | A7A1_0231 | AGA20849.1 |
| 115 | *Bacillus subtilis subsp. subtilis str. JH642 substr. AG174* | BSUA_01330 (YjiC) | AIC39616.1 |
| 116 | *Bacillus subtilis subsp. subtilis str. OH 131.1* | Q433_06955 | AIC97767.1 |
| 117 | *Bacillus subtilis subsp. subtilis str. RO-NN-1* | I33_1374 | AEP90343.1 |
| 118 | *Bacillus subtilis SX01705* | BSSX_1367 | ASK23262.1 |
| 119 | *Bacillus subtilis SZMC 6179J* | A3772_06685 | AMS46762.1 |
| 120 | *Bacillus subtilis T30* | BIS30_16840 | AJW86673.1 |
| 121 | *Bacillus subtilis TO-A* | M036_06410 | AII35227.1 |
| 122 | *Bacillus subtilis TO-A JPC* | ABU16_2251 | AKN13327.1 |
| 123 | *Bacillus subtilis UD1022* | ABA10_06695 | AKI91665.1 |
| 124 | *Bacillus subtilis VV2* | BKN48_16640 | AOY06829.1 |
| 125 | *Bacillus subtilis XF-1* | C663_1253 (YjiC) | AGE63073.1 |
| 126 | *Bacillus vallismortis NBIF-001* | B9C48_06160 | ARM27416.1 |
| 127 | *Bacillus velezensis 157* | CFN60_06275 | ASK58003.1 |
| 128 | *Bacillus velezensis 9D-6* | B7941_08800 | ARJ76689.1 |
| 129 | *Bacillus velezensis AS43.3* | B938_06275 | AFZ90281.1 |
| 130 | *Bacillus velezensis B25* | BAMMD1_1150 (PpuG) | CUX93057.1 |
| 131 | *Bacillus velezensis CAU B946* | BACAU_1181 (YjiC) | CCF04715.1 |
| 132 | *Bacillus velezensis CBMB205* | BCBMB205_12130 | ANF36113.1 |
| 133 | *Bacillus velezensis CC09* | A1D33_003205 | ANB46347.1 |
| 134 | *Bacillus velezensis D2-2* | A2I97_05855 | AOU00612.1 |
| 135 | *Bacillus velezensis FZB42* | RBAM_012300 | ABS73593.1 |
| 136 | *Bacillus velezensis G341* | ABH13_1234 | AKL75824.1 |
| 137 | *Bacillus velezensis GH1-13* | BVH55_06525 | AQS43570.1 |
| 138 | *Bacillus velezensis GQJK49* | BAGQ_1311 | ARZ57545.1 |
| 139 | *Bacillus velezensis JJ-D34* | AAV29_06045 | AKF30116.1 |
| 140 | *Bacillus velezensis JS25R* | NG74_01252 (Oled_2) | AIU81354.1 |
| 141 | *Bacillus velezensis JTYP2* | BAJT_06155 | ARB32876.1 |
| 142 | *Bacillus velezensis LS69* | A8142_05880 | ANU29702.1 |
| 143 | *Bacillus velezensis M75* | BBJ33_06435 | AOO61172.1 |
| 144 | *Bacillus velezensis NAU-B3* | BAPNAU_2561 (YjiC) | CDH96341.1 |
| 145 | *Bacillus velezensis NJN-6* | AW02_012410 | AKD29392.1 |
| 146 | *Bacillus velezensis S3-1* | A5891_06015 | ANS37942.1 |
| 147 | *Bacillus velezensis SB1216* | A6R78_04970 | ANB85897.1 |
| 148 | *Bacillus velezensis SCDB 291* | CHN56_00937 (Tylcv_1) | ASS61481.1 |
| 149 | *Bacillus velezensis SQR9* | V529_11730 | AHZ15199.1 |
| 150 | *Bacillus velezensis SRCM100072* | S100072_01351 | ASB52687.1 |
| 151 | *Bacillus velezensis SRCM101413* | S101413_01476 (fragment) | ASB64923.1 |
| 152 | *Bacillus velezensis sx01604* | BLL65_12625 | AQZ73791.1 |
| 153 | *Bacillus velezensis T20E-257* | CEG11_06350 | ASF54731.1 |
| 154 | *Bacillus velezensis TB1501* | CG798_11870 | ASP27404.1 |
| 155 | *Bacillus velezensis TrigoCor1448* | AJ82_06895 | AHK48771.1 |
| 156 | *Bacillus velezensis UCMB5033* | RBAU_1185 (YjiC) | CDG29153.1 |
| 157 | *Bacillus velezensis UCMB5036* | BAM5036_1136 (YjiC) | CCP21183.1 |
| 158 | *Bacillus velezensis UCMB5113* | BASU_1164 (YjiC) | CDG25461.1 |
| 159 | *Bacillus velezensis YAU B9601-Y2* | BANAU_1162 (YjiC) | CCG49183.1 |
| 160 | *Bacillus velezensis YJ11-1-4* | AAV30_13175 | AKF77053.1 |
| 161 | *Bacillus subtilis JRS11* | Oleandomycin glycosyltransferase | CUB50191.1 |

**Table S2.** Amino acid identity among the ten selected GTs.

| **GTs (%)** | **YjiC** | **BsGT-1** | **BgGT** | **BaGT** | **BpGT** | **BssGT** | **BamGT** | **YojK** | **BcGT-1** |
| --- | --- | --- | --- | --- | --- | --- | --- | --- | --- |
| **BsGT-1** | 56.1 |  |  |  |  |  |  |  |  |
| **BgGT** | 75.2 | 57.3 |  |  |  |  |  |  |  |
| **BaGT** | 54.3 | 77.0 | 55.1 |  |  |  |  |  |  |
| **BpGT** | 94.4 | 55.6 | 76.0 | 54.6 |  |  |  |  |  |
| **BssGT** | 55.1 | 88.0 | 55.8 | 74.8 | 54.8 |  |  |  |  |
| **BamGT** | 53.3 | 59.6 | 54.3 | 57.6 | 53.8 | 58.3 |  |  |  |
| **YojK** | 28.6 | 30.1 | 30.0 | 28.1 | 29.3 | 29.1 | 29.1 |  |  |
| **BcGT-1** | 30.6 | 31.1 | 32.0 | 29.3 | 30.6 | 30.6 | 29.3 | 41.7 |  |
| **BsGT-2** | 27.4 | 28.9 | 25.7 | 28.7 | 26.4 | 27.4 | 26.7 | 32.0 | 32.5 |

**Table S3.** 1D NMR (600 MHz, CD_3_OD) data for epothilone 7-*O*-β-D glucoside ^a^

| Pos. | *δ*_H_ (mult., *J* (Hz)) | *δ*_C_ | Pos. | *δ*_H_ (mult., *J* (Hz)) | | *δ*_C_ |
| --- | --- | --- | --- | --- | --- | --- |
| 1 | − | 172.0 | 17 | | 6.60 (s) | 120.3 |
| 2 | 2.58 (m, 2H) | 40.1 | 18 | | − | 153.2 |
| 3 | 4.28 (dd, *J* = 8.6, 5.4 Hz, 1H) | 73.0 | 19 | | 7.24 (s) | 117.7 |
| 4 | − | 54.6 | 20 | | − | 167.0 |
| 5 | − | 220.5 | 21 | | 2.69 (s, 3H) | 18.7 |
| 6 | 3.47 (m, 1H) | 46.3 | 22 | | 1.05 (s, 3H) | 21.2 |
| 7 | 3.81 (dd, *J* = 6.0, 1.8 Hz, 1H) | 85.0 | 23 | | 1.30 (s, 3H) | 22.0 |
| 8 | 1.72 (m, 1H) | 38.9 | 24 | | 1.29 (d, *J* = 7.2 Hz, 3H) | 16.4 |
| 9 | 1.61 (m, 1H);  1.33 (m, 1H) | 31.8 | 25 | | 1.04 (d, *J* = 7.2 Hz, 3H) | 17.7 |
| 10 | 1.62 (m, 1H);  1.42 (m, 1H) | 25.6 | 26 | | 2.07 (s, 3H) | 15.7 |
| 11 | 1.67 (m, 1H);  1.55 (m, 1H) | 27.8 | 1' | | 4.46 (d, *J* = 7.8 Hz, 1H) | 104.8 |
| 12 | 2.92 (dt, *J* = 7.8, 3.6 Hz, 1H) | 58.8 | 2' | | 3.22 (t, *J* = 7.8 Hz, 1H) | 75.8 |
| 13 | 3.09 (dt, *J* = 8.4, 4.2 Hz, 1H) | 55.7 | 3' | | 3.37 (t, *J* = 9.0 Hz, 1H) | 78.3 |
| 14 | 2.09 (m, 1H);  1.96 (m, 1H) | 32.9 | 4' | | 3.32 (m, 1H) | 71.8 |
| 15 | 5.45 (dd, *J* = 7.8, 1.2 Hz, 1H) | 77.7 | 5' | | 3.28 (m, 1H) | 77.7 |
| 16 | − | 139.4 | 6' | | 3.86 (dd, *J* = 12.0, 1.8 Hz, 1H);  3.69 (dd, *J* = 12.0, 5.4 Hz, 1H) | 62.9 |

^a^ our isolated epothilone A 7-O-β-D glucoside was recorded in CD_3_OD, which is consistent with the reference. The carbon numbering is shown in Figure 1.
